# Harnessing phosphonate antibiotics argolaphos biosynthesis enables a synthetic biology-based green synthesis of glyphosate

**DOI:** 10.1038/s41467-022-29188-6

**Published:** 2022-04-01

**Authors:** Leixia Chu, Xiaoxia Luo, Taoting Zhu, Yingying Cao, Lili Zhang, Zixin Deng, Jiangtao Gao

**Affiliations:** 1 State Key Laboratory of Ecological Pest Control for Fujian and Taiwan Crops, College of Life Sciences, Fujian Agriculture and Forestry University, 350002 Fuzhou, China; 2 Key Laboratory of Biopesticide and Chemical Biology of Ministry of Education, College of Life Sciences, Fujian Agriculture and Forestry University, 350002 Fuzhou, China; 3 Xinjiang Production and Construction Corps Key Laboratory of Protection and Utilization of Biological Resources in Tarim Basin, College of Life Science & Technology, Tarim University, Alar, Xinjiang, 843300 China; 4 State Key Laboratory of Microbial Metabolism, Joint International Laboratory on Metabolic and Developmental Sciences, School of Life Sciences and Biotechnology, Shanghai Jiao Tong University, 800 Dongchuan Road, Shanghai, 200240 China

**Keywords:** Metabolic engineering, Biocatalysis, Applied microbiology, Synthetic biology

## Abstract

Glyphosate is a widely used herbicide with an annual production of more than one million tons globally. Current commercialized production processes of glyphosate are generally associated with manufacturing hazards and toxic wastes. Recently, many countries have strengthened environmental supervision and law enforcement on glyphosate manufacturing. Therefore, a green source of glyphosate is required. Here, we characterize the genes required for producing aminomethylphosphonate (AMP), one of the intermediates in the biosynthesis of the potent antibiotics argolaphos. We apply a synthetic biology strategy to improve AMP production in *Streptomyces lividans*, with fermentation titers of 52 mg L^-1^, a 500-fold improvement over the original strain. Furthermore, we develop an efficient and practical chemical process for converting AMP to glyphosate. Our findings highlight one greenness-driven alternative in the production of glyphosate.

## Abstract (web summary)

Current commercialized production processes of glyphosate are generally associated with manufacturing hazards and toxic wastes. Here, to address the problems, the authors report the bioproduction of aminomethylphosphonate (AMP) and a chemical process for the conversion of AMP to glyphosate.

## Introduction

Glyphosate (*N*-(phosphonomethyl)glycine, **1**) is a globally used herbicide for weed control. Since the introduction of glyphosate to the commercial market under the trade name Roundup^®^ by Monsanto in 1974, numerous glyphosate-containing formulations have vastly changed agricultural structures globally and freed people from manual weeding^1^. Although numerous legislation regulations and banning of glyphosate for residential use have been introduced due to its carcinogenic activity^2^, glyphosate is still one of the leading and fastest-growing agrochemicals in the world.

Two main approaches are widely used to synthesize glyphosate industrially (Supplementary Fig. 1)^3,4^. The first one is based on iminodiacetic acid (IDA). The second one involves hydrophosphonylation using dimethyl phosphate (DMP) in a one-pot synthesis. Although these approaches can stably produce glyphosate in high yield, they either use toxic reagents and solvents, produce a great deal of environmentally harmful wastes, lead to difficulties in waste processing and recycling, or require high investments in equipment and instruments. For example, chlorine, formaldehyde, hydrochloric acid, methanol, phosphorus, triethylamine, and cyanide compounds, belonging to the harmful environmental pollutants, are among 189 hazardous environmental pollutants specified in the new Clean Air Act by the US Congress^5^. Therefore, more investments are needed to set up the processing and recycling equipment for these toxic chemicals and wastes.

Furthermore, the global production of glyphosate has reached more than one million tons annually. Its global demand probably will not be significantly affected by the recent banning on residential use due to the continued expansion of transgenic crop cultivation^6^. Many countries are strengthening environmental supervision on high-polluting businesses, including the glyphosate-related industry, so many manufacturers have quited business in recent years. Therefore, to meet the demand for glyphosate, a green and efficient process for producing glyphosate is urgently needed.

One of the most widely used strategies to perform these chemical processes in a more environmentally friendly fashion is the application of biocatalyst, which refers to the use of biological systems or their parts to speed up chemical reactions. As Nature has evolved on advancing a great many chemical aspects to establish limitlessly complicated structures in cellular metabolites, biological processes can produce optically pure precursors with good chemoselectivity, regioselectivity, and stereoselectivity for chemical syntheses of high-value chemicals widely used in medicine and agriculture^7^. Microbially-based production platforms solve many of those problems that occurred in chemical synthesis and can transform the manufacturing processes for many bio-based chemicals^8^. Advancements in next-generation DNA sequencing and bioinformatics have accelerated the elucidation of complex biochemical pathways. They can now be easily expressed in genetically tractable hosts for scalable industrial production. Meanwhile, the rapid advancement of powerful synthetic biology tools for genetically refactoring metabolic pathways or manipulating microorganism cells has considerably enhanced the production of bio-based chemicals. Commercial production of artemisinic acid^9^ in the yeast *Saccharomyces cerevisiae* and opiates in the engineered bacterium *Escherichia coli*^10^ or the yeast *S. cerevisiae*^11^ are trailblazing examples of these revolutionary technologies invigorating chemical manufacturing.

Genetic elements, including promoters and ribosome-binding sites (RBSs), affect the transcription and translation of their downstream genes, respectively. In addition, the regulatory elements could interfere with each other by the promoter clearance and escape or by forming some special structures unreachable to ribosomes on the mRNA. To address this problem, various insulators have been characterized to break the unexpected regulatory element interaction^12–14^.

**Fig. 1:**
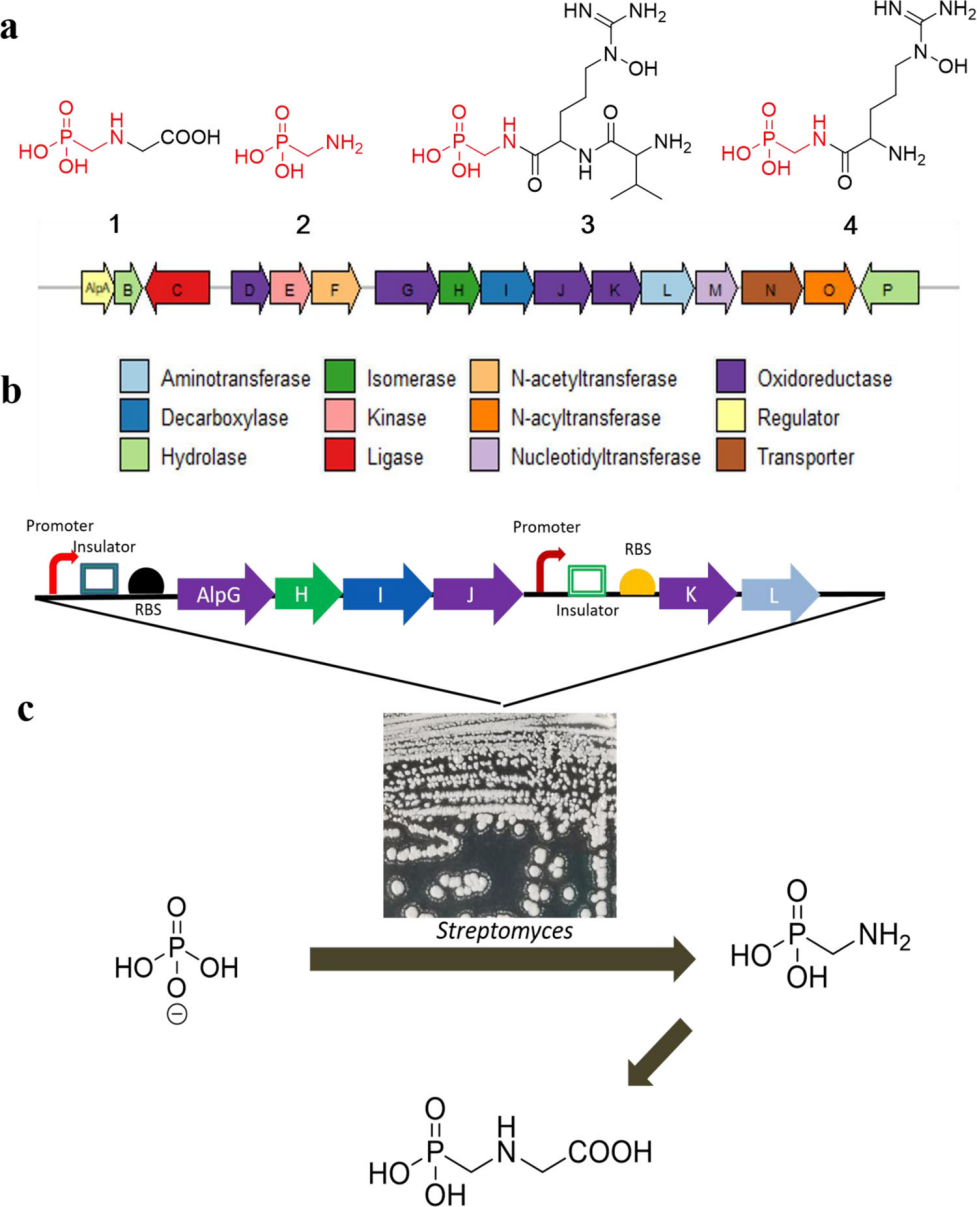
A synthetic biology-based semisynthesis of the herbicide glyphosate (1). **a** Chemical structures of glyphosate (**1**), aminomethylphosphonate (AMP) (**2**), argolaphos A (**3**), and argolaphos B (**4**). **b** DNA fragments from *S. monomycini* NRRL B-24309 that contain the gene *alpH*, the homolog of *pepM* gene. Genes are color-coded according to their putative function according to the analysis in Table 1. **c** The workflow of the synthetic biology strategy used to produce **1** from inorganic phosphates.

As glyphosate is not naturally synthesized, it is currently unlikely to produce this herbicide by fermentation. In 2015, we reported two phosphonopeptides, argolaphos A and B (**3** and **4**), isolated from spent media of *S. monomycini* NRRL B-24309^15^. Both compounds are composed of aminomethylphosphonate (AMP, **2**) in a peptide bond with a non-standard amino acid *N*^5^-hydroxyarginine (Fig. 1a). AMP (**2**) has previously been confirmed as a shunt metabolite in a *ΔphpJ* mutant (*phhJ* encodes an aldehyde dehydrogenase) of the phosphinothricin*-*producing strain *S. hygroscopicus* ATCC 21705^16^ or a significant product as glyphosate decomposes^17^.

Here, we report an efficient and eco-friendly methodology for the production of glyphosate (Fig. 1c). First, we identify the genes required for producing aminomethylphosphonate (AMP). Then, we use a synthetic biology strategy to improve AMP production in *S. lividans*. Next, we establish an efficient and practical chemical process for converting AMP to glyphosate. Our methodology may enhance the provision of first-line weed control with less environmental impact and at a reduced price.

## Results and discussion

### Determination of absolute configuration

**Fig. 2:**
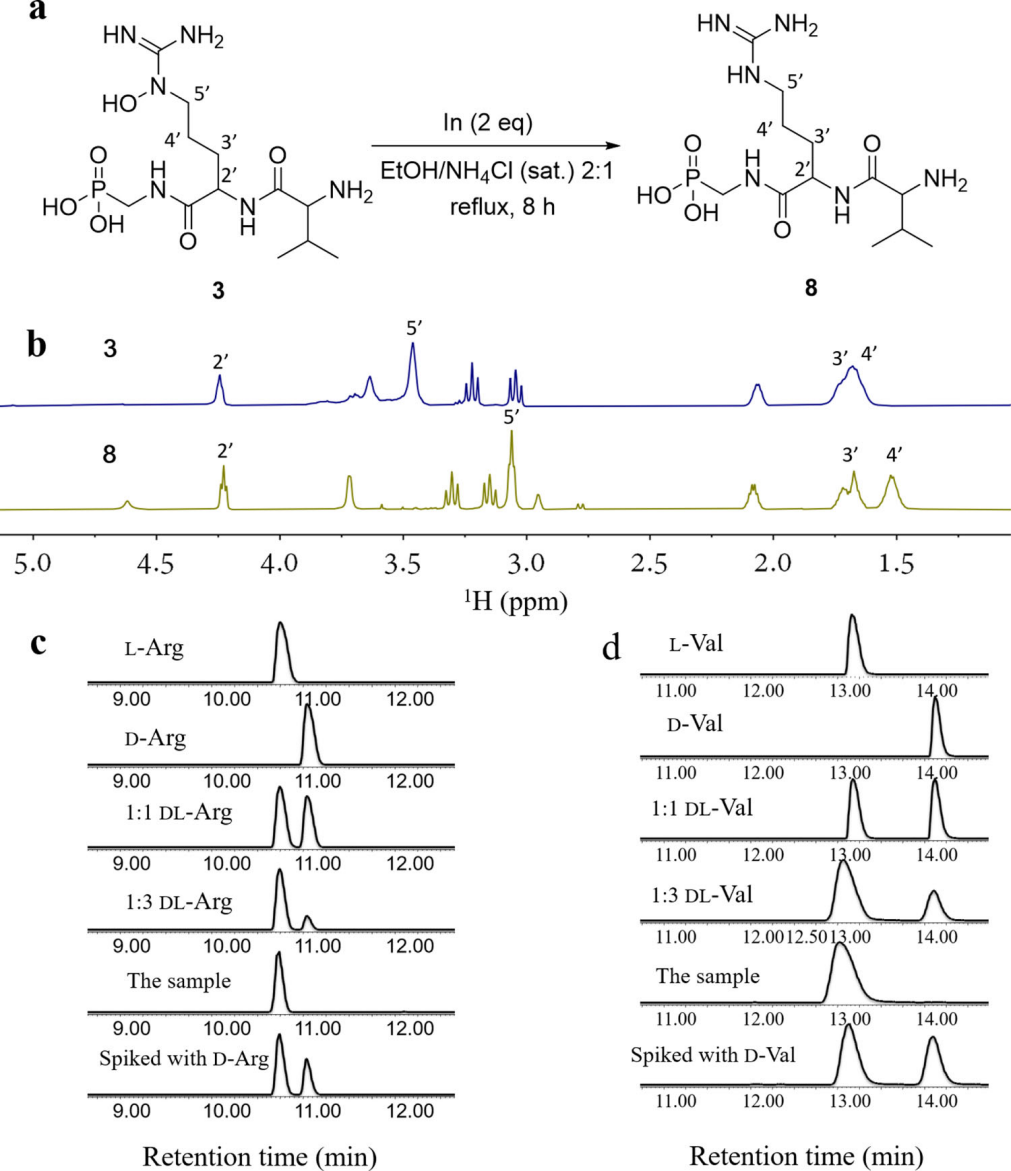
Determination of absolute configuration of argolaphos A (3). **a** Indium-mediated reduction of N^5^-hydroxyarginine in **3** led to compound **8**. **b** Comparison of ^1^H NMR spectrum of **3** and **8**. **c** Marfey analysis for determination of the absolute configuration of arginine in **8**. LC-HRMS chromatograms were monitored at m/z = 427.160 for the *N*-(5-fluoro-2,4-dinitrophenyl)-d-alaninamide (FDAA) derivatives of standard l-arginine, d-arginine, dl-arginine, the sample, and the sample spiked with d-arginine. **d** Marfey analysis for determination of the absolute configuration of valine in **8**. LC-HRMS chromatograms were monitored at m/z = 370.136 for the *N*-(5-fluoro-2, 4-dinitrophenyl)-d-alaninamide (FDAA) derivatives of standard l-valine, d-valine, dl-valine, the sample, and the sample spiked with d-valine.

As the planar structure of **3** was elucidated^15^, we need to determine the absolute configuration of **3**, which contains two amino acids, *N*^5^-hydroxyarginine, and valine. We carried out acid hydrolysis and derivatization of the hydrolysates with Marfey’s reagent D-FDAA (*N*-(5-fluoro-2, 4-dinitrophenyl)-d-alaniamide). Some groups showed that the hydroxy group of *N*^5^-hydroxyarginine was removed under acidic conditions^18,19^. However, in our case, we could not detect the product of arginine after **3** was hydrolyzed in 6 N HCl at 120 °C for 12 h. To effectively accomplish the reductive cleavage of the N-O bond in **3**, we employed a variety of reducing reagents, including Zn/HCl^20^, H_2_/Pd^21,22^, TiCl_3_^23^, Zn/Cu(OAc)_2_/AcOH^24^, NaBH_4_-NiCl_2_^25^, and indium (In(0))^26^. We found that indium metal with a mixed ethanol/saturated aqueous NH_4_Cl solution (2:1) gave the reduced product **8** with a high yield of 95% (Fig. 2a). The chemical structure of **8** was confirmed using ^1^H, ^13^C, ^31^P NMR spectra, and high-resolution mass spectrometry (HRMS) data (Fig. 2b, Supplementary Figs. 2–5, and Supplementary Table 2). Then the absolute configuration of arginine in **8** was established to be l using advanced Marfey’s method (Fig. 2c). By comparing the retention times from LC/MS analysis of the D-FDAA derivatives^27^ with the same reaction products of authentic l and d valines, the absolute configuration of valine in **3** was determined to be l (Fig. 2d).

### Genome mining approach to gene cluster discovery and validation

Biosynthesis of most C-P-containing compounds starts with the same first reaction catalyzed by the enzyme phosphoenolpyruvate (PEP) mutase (encoded by *pepM*). Furthermore, the *pepM* gene from various microorganisms can be easily obtained with degenerate PCR primers. The *pepM*-based genoming method has been extensively used to screen a collection of approximately 10,000 actinomyces, leading to 278 *pepM*^+^ strains, including the *S. monomycini* NRRL B-24309^15^. The *S. monomycini* NRRL B-24309 genome was sequenced and then annotated utilizing Rapid Annotation Using Subsystem Technology (RAST)^28^. Extensive analysis of biosynthetic gene clusters using antiSMASH^29^ led to the location of a unique PEP mutase-encoding gene cluster at an approximately 17.6 kbp locus in the whole genome. The putative *alp* gene cluster comprises 16 open reading frames (ORFs) in the 17.6 kbp locus. The *alp* gene cluster is shown schematically in Fig. 1b, and all ORFs from this gene cluster are listed in Table 1 along with their predicted functions.

**Table 1:** Summary of open reading frames in the genomic DNA of *S. monomycini* NRRL B-24309 that includes the argolaphos biosynthetic gene cluster.

ORF	No. of residues	Highest scoring conserved domain hits (accession number)	Proposed functions	E-value
AlpA	202	COG2197	DNA-binding response regulator	1.29e-25
AlpB	195	PRK15393	NUDIX hydrolase	5.31e-41
AlpC	416	PRK02186	ATP-grasp ligase	2.35e-18
AlpD	274	pfam08892	YqcI/YcgG family	2.44e-58
AlpE	291	cd04413	Nucleoside diphosphate kinase	5.11e-12
AlpF	342	pfam13480	GNAT N-acetyltransferase	1.08e-07
AlpG	444	cd20489	Hydroxypropylphosphonic acid epoxidase	5.39e-32
AlpH	290	TIGR02320	Phosphoenolpyruvate mutase	3.30e-96
AlpI	379	TIGR03297	Phosphonopyruvate decarboxylase	5.16e-112
AlpJ	402	cd08182	Phosphonoacetaldehyde reductase	3.42e-100
AlpK	348	cd05299	Dehydrogenase	3.44e-98
AlpL	374	PRK05764	Aminotransferase	1.79e-80
AlpM	291	cd05403	Nucleotidyltransferase	5.63e-06
AlpN	438	cd06173	Major facilitator superfamily (MFS)	8.83e-33
AlpO	356	COG3146	GNAT N-acyltransferase	5.24e-07
AlpP	415	pfam00144	β-lactamase	1.76e-43

*ORF* open reading frame.

**Fig. 3:**
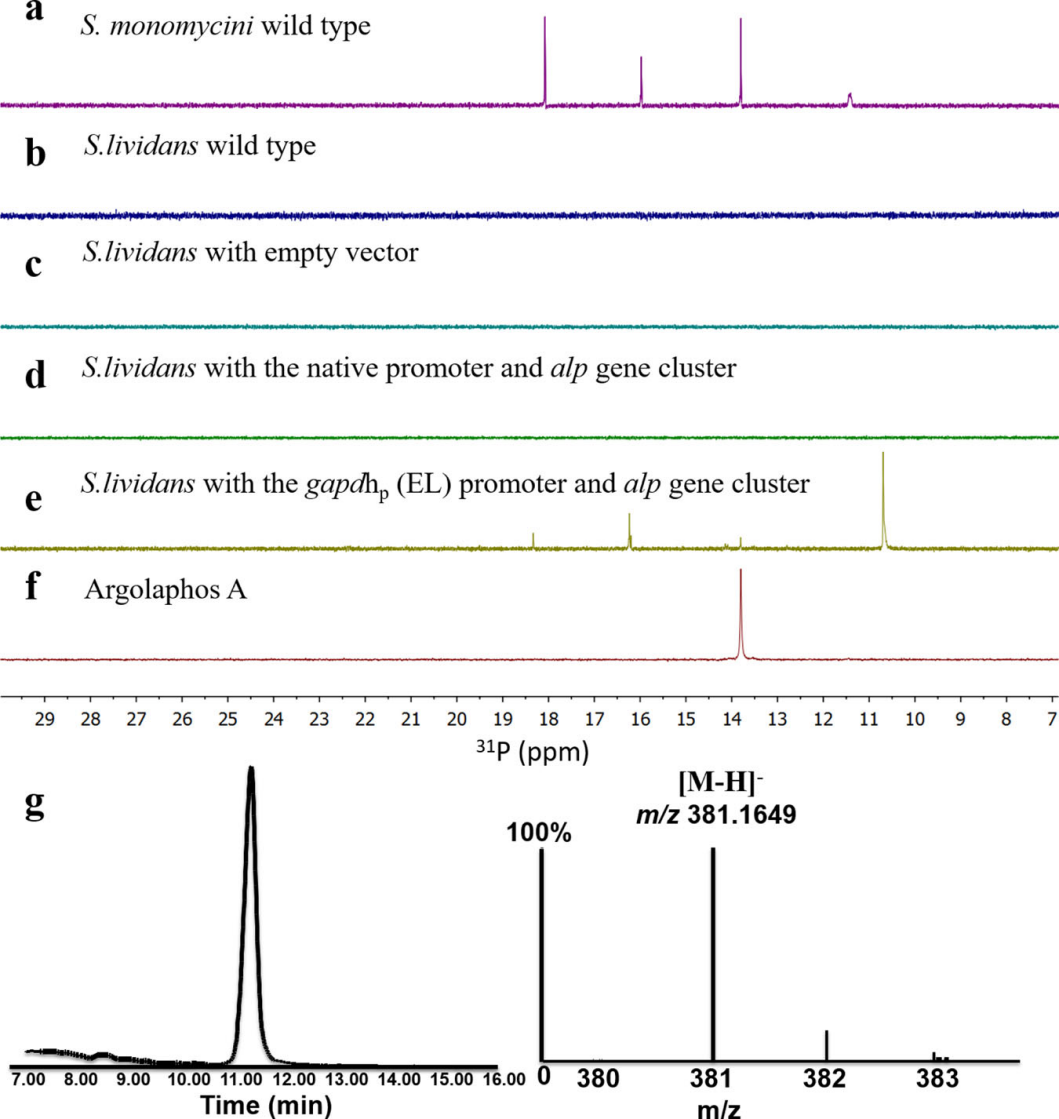
Analysis of the full-cluster integrated strain *S. lividans* 66. **a** ^31^P NMR spectrum of the culture extracts from *S. monomycini* NRRL B-24309. **b** ^31^P NMR spectrum of the wild-type *S. lividans* 66. **c** ^31^P NMR spectrum of *S. lividans* 66 transformed with empty vector. **d** ^31^P NMR spectrum of *S. lividans* 66 transformed with the native promoter and *alp* gene cluster. **e** ^31^P NMR spectrum of *S. lividans* 66 transformed with the *gapdh*_p_ (*E. lenta*, EL) promoter and *alp* gene cluster. **f** ^31^P NMR spectrum of argolaphos A standard. **g** Extracted ion current chromatogram of the culture extracts from *S. lividans* 66 transformed with the *gapdh*_p_ (*E. lenta*, EL) promoter and *alp* gene cluster.

Initially, we failed to knock out any genes in the *alp* gene cluster from *S. monomycin* NRRL B-24309 by both double crossover-mediated and CRISPR-Cas9-based deletion^30^. Therefore we had to express the *alp* gene cluster in a genetically tractable host *S. lividans* 66 to confirm and characterize the *alp* gene cluster. DNA assembler was used to construct the pYES plasmids carrying the *alp* cluster (*alpA*-*P*) with different strong well-characterized promoters previously reported^31^. Pathway fragments of the *alp* cluster and helper fragments containing the genetic elements required for DNA maintenance and replication in *S. cerevisiae*, *E. coli*, and *S. lividans* were PCR-amplified from the purified genomic DNA of the original producer *S. monomycin*, and the two corresponding vectors pRS416, pAE4, respectively^31,32^. All DNA fragments were assembled into a recombinant vector in *S. cerevisiae* using homologous recombination, and the isolated plasmids were electroporated into *E. coli* for their enrichment and further verification. Finally, the correct construct was integrated into *S. lividans* 66 for heterologous expression of the *alp* gene cluster. When we constructed the intact regulatory elements (the original *alp* gene cluster with the native promoter and RBS, Supplementary Fig. 8), no production of **3** was found in the culture extract (Fig. 3a–d). As the *gapdh*p promoter from the bacillus *Eggerthella lenta* is particularly active under laboratory culturing conditions, we constructed the plasmid transformed with the *gapdh*_p_ (*E. lenta*) promoter added upstream of the whole *alp* cluster (Supplementary Fig. 8). The production of a small amount of **3** was confirmed by ^31^P NMR and LC-MS (Fig. 3e–g). Altogether, the heterologous expression of the *alp* cluster strongly implicates a direct role of the *alp* cluster in the biosynthesis of **3**.

### Proposed biosynthesis pathway

Both **3** and **4** were first isolated from *S. monomycin* NRRL B-24309 due to a high-throughput screening program focused on new antibiotics^15^. The two phosphonopeptide congeners differ in the number of amino acids. Compound **4** consists of **2** in a peptide bond with the nonproteinic amino acid *N*^5^-hydroxy-l-arginine, whereas **3** has an additional amide linkage with l-valine. We isolated **2** from the culture extract of the original strain *S. monomycini* NRRL B-24309. Therefore we believe that **2** is the intermediate in the biosynthesis of **3** and **4**.

**Fig. 4:**
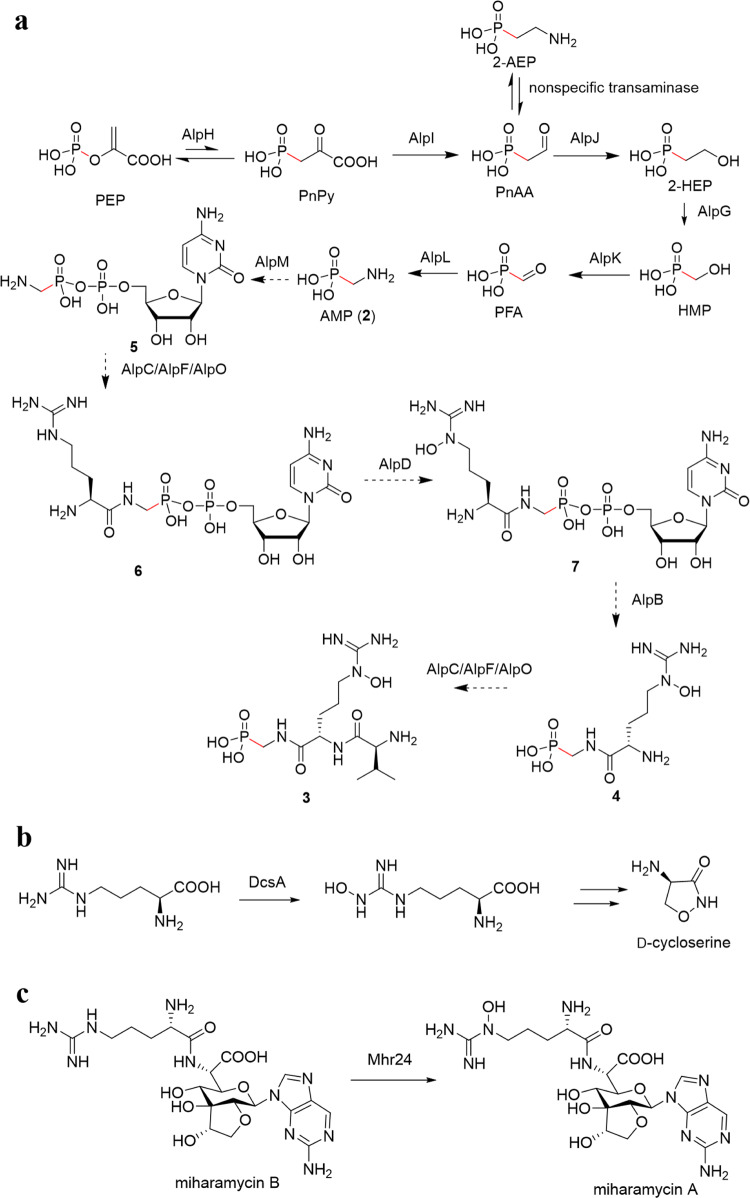
Biosynthesis of the phosphonate antibiotic argolaphos A (3). **a** Proposed biosynthetic pathway of argolaphos A (**3**). Putative steps are indicated with dashed arrows. PEP phosphoenolpyruvate, PnPy phosphonopyruvate, PnAA phosphonoacetaldehyde, 2-HEP 2-hydroxyethylphosphonate, HMP hydroxymethylphosphonate, PFA phosphonoformaldehyde. **b** DcsA is the proposed hydroxylase involved in arginine *N*^ω^-oxidation in the biosynthesis of d-cycloserine. **c** Mhr24 is a hydroxylase in the oxidation of arginine *N*^5^ of miharamycin B to yield miharamycin A.

An extensive inspection of the *alp* gene cluster of *S. monomycini* NRRL B-24309 indicated a possible biosynthetic pathway for the production of **3** (Fig. 4a). The biosynthesis of **2** involves homologs of key enzymes PhpA–PhpE in the phosphinothricin biosynthesis^15,16^. AlpH is the PEP mutase that catalyzes the rearrangement of PEP to phosphonopyruvate (PnPy). AlpI catalyzes the decarboxylation of PnPy to yield phosphonoacetaldehyde (PnAA) which is then reduced to 2-HEP, catalyzed by the Fe(III)-dependent alcohol dehydrogenase AlpJ with NADH as a cofactor.

AlpG is a mononuclear nonheme iron enzyme similar in sequence to hydroxypropylphosphonic acid epoxidase (HppE)^33^, hydroxyethylphosphonate dioxygenase (HEPD)^34^, and methylphosphonate synthase (MPnS)^35^(Supplementary Tables 3, 4 and Supplementary Figs. 6, 7). Phylogenetic analysis showed that AlpG belongs to class II HEPD as it contains a Tyr^158^-G^179^ motif, which is diagnostic of class II HEPD (Supplementary Figs. 6, 11). All the above indicate that the protein AlpG might catalyze the formation of hydroxylmethylphosphonate (HMP) from 2-HEP in the biosynthesis of **3** (Fig. 4a). As AlpK possesses sequence similarity to alcohol dehydrogenases, the conversion catalyzed by AlpK would be the reduction of HMP to phosphonoformaldehyde (PFA). PFA is then transaminated to produce **2**, catalyzed by the pyridoxal phosphate-dependent aminotransferase AlpL.

While the *N*-hydroxyguanidine moiety of the *N*^5^-hydroxy-l-arginine shown in **3** is highly similar to an intermediate in a five-electron oxidative reaction of l-arginine to l-citrulline catalyzed by nitric oxide synthase (NOS), no homolog of NOS is found in the *alp* gene cluster. Although *N*^5^-hydroxy-l-arginine and similar hydroxylated guanidines have been previously reported in the field of natural products^36–40^, the biosynthesis of this interesting moiety is still a mystery. Only a few enzymes responsible for *N*-hydroxylation of l-arginine are identified and studied. DcsA, a “YqcI/YcgG” protein, is involved in the hydroxylation of l-arginine to *N*^ω^-hydroxy-l-arginine in the biosynthesis of d-cycloserine produced by the strain *S. lavendulae* ATCC 11924 (Fig. 4b)^41^. Mhr24, a “YqcI/YcgG” homolog, is a putative hydroxylase involved in the biosynthesis of *N*^5^-hydroxy-l-arginine found in the nucleoside antibiotic miharamycin A from the strain *S. miharaensis* ATCC 19440 (Fig. 4c)^42^. We found that AlpD exhibits moderate sequence homology (24.7% similarity and 17.7% identity) to Mhr24. AlpD appeared as an acceptable candidate for catalyzing the required *N*-hydroxylation in the formation of *N*^5^-hydroxy-l-arginine in the biosynthesis of **3**.

The biosynthesis of **3** involves incorporating two amino acids *N*^5^-hydroxy-l-arginine and l-valine, into the final structure so that two peptide bonds are required between amines and carboxylic acid. Interestingly, the *alp* gene cluster encodes putative amidoligases belonging to three different protein families. For example, AlpC contains an ATP-grasp domain^43^. Both AlpF and AlpO, members of the GCN5-like *N*-acetyltransferase (GNAT) superfamily, serve as peptide aminoacyl tRNA ligases^44^. More experiments are needed to decide which of these three enzymes are responsible for those two amide bonds shown in Fig. 4a.

The gene cluster also provides clues on the possible assembly of **4** from **2**. The intermediate **2** is likely subjected to cytidylyl activation catalyzed by the enzyme AlpM to produce the cytidine monophosphate (CMP)-conjugate **5**. Cytidylyl activation has been well documented in the biosynthesis of cell surface glycans, phospholipids, and phosphonate natural products^45^. Similar enzymes have been identified in the biosynthesis of several phosphonates, including fosfomycin, FR-900098, and phosphinothricin tripeptide (PTT)^46^. The PepM enzyme Fom1 in the biosynthesis of fosfomycin possesses its *C*-terminal PepM domain and its *N*-terminus cytidylyltransferase (CyTase) domain. The latter catalyzes the conjugation of 2-HEP with cytidine triphosphate (CTP) to yield HEP-CMP^47^. FrbH, a bifunctional enzyme with an *N*-terminal cytidylyltransferase domain and a *C-*terminal decarboxylase domain. The former catalyzes the cytidylylation of 2-amino-4-phosphonobutyrate, and the latter is responsible for the subsequent decarboxylation to yield CMP-3-aminobutylphosphonate^48^. In the biosynthesis of PTT, the nucleotidylyltransferase homolog PhpF catalyzes the displacement of the pyrophosphate of CTP by phosphonoformate to furnish the intermediate CMP-5′-phosphonoformate (CMP-5′-PF)^16^. All above illuminate cytidylyl activation as a ubiquitous chemical logic in the biosynthesis of C-P containing natural products and pave the way for probing diverse tailoring pathways^45^. Therefore, we propose that the nucleotidylyltransferse AlpM might activate **2** to furnish AMP-CMP as a suitable substrate for peptide bond formation.

### Biosynthesis of AMP

As discussed above, six genes *alpGHIJKL* are assumed to be required for the biosynthesis of **2**. We moved on to confirm the minimum genetic cassette of the biosynthesis of **2** and determine the tolerance of one gene or set of genes to changes in expression levels. First, we reconstructed the entire six-gene gene cluster *alpG-L* by inserting the strong promoter ermE*p upstream of this gene cluster. One control carrying the empty vector was also constructed. Second, to decipher the biosynthetic steps of **2**, a series of gene combinations were built (Supplementary Fig. 9). All these constructs were integrated into *S. lividans* 66 for heterologous expression. We then extracted the metabolites from the *S. lividans* 66 harboring the reconstructed *alpG-L* gene cluster and performed ^31^P NMR analysis.

**Fig. 5:**
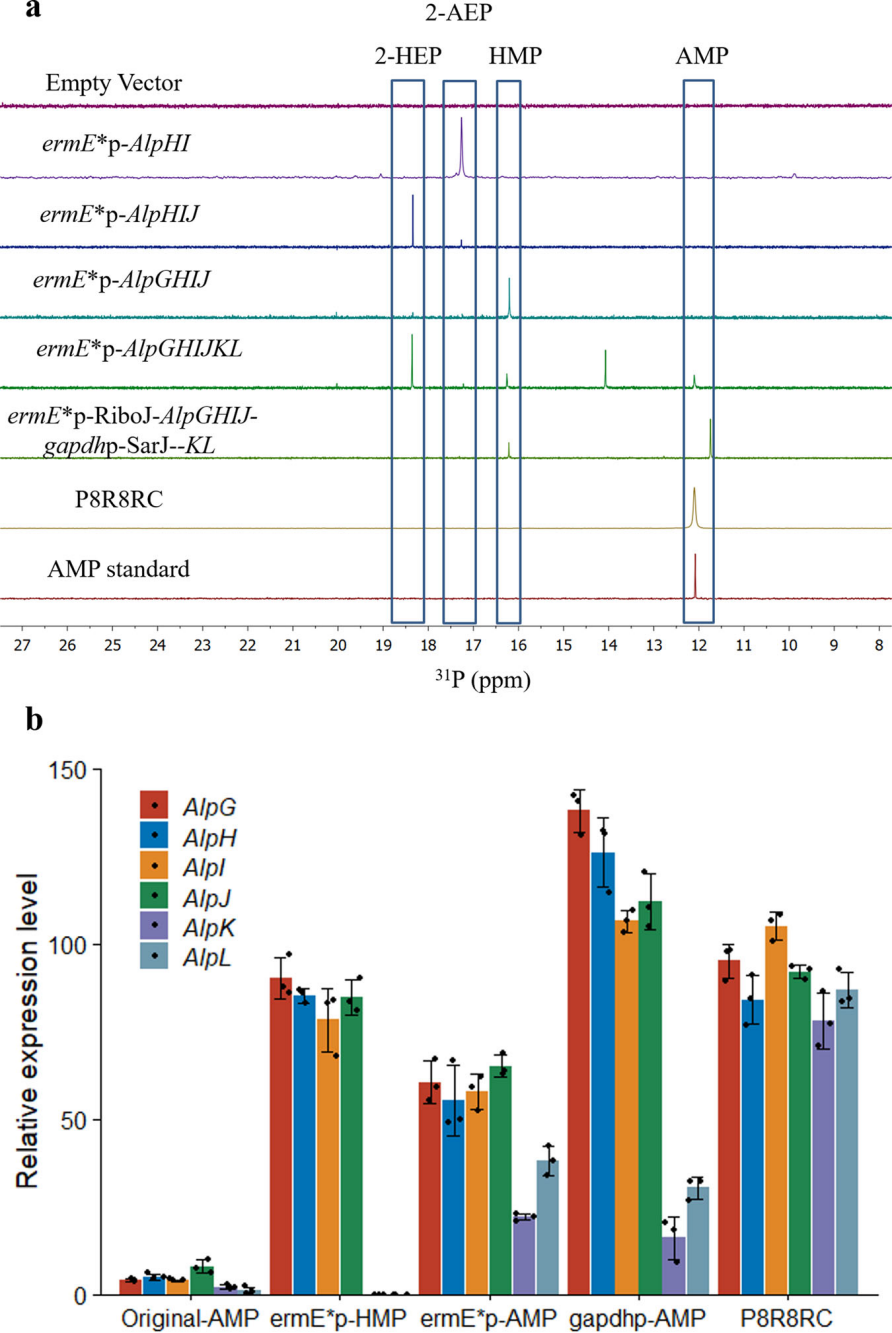
Production of AMP in *S. lividans*. **a** ^31^P NMR spectrum of production profiles of *S. lividans* 66 transformed with combinations of *alpGHIJKL*. **b** Real-time quantitative PCR analysis of AMP genes in the reconstructed gene cluster and the control gene cluster. AMP amonomethylphosphonate, RC reconstructed cluster. Data were represented as mean ± SD (*n* = 3 independent experiments). Source data are provided as a Source Data file.

Chemical shifts in ^31^P NMR are easily affected by the concentration of the compound of interest, the solvent used, and the occurrence of impurities as the external standard 85% phosphoric acid can not fully consider the whole properties of the sample. Therefore, ^31^P chemical shifts for the same compound in different contexts could fluctuate by 1 ppm or more, particularly for phosphate or phosphonate groups (*P* = O)^49^. In our study, AMP’s chemical shifts ranged from 11.6 to 12.1 ppm in different samples (Fig. 5a). As shown in Fig. 5a, the host strain carrying six genes *alpG-L* produced the compound with a strong resonance (δ 11.6 ppm) corresponding to **2**. The production of **2** was low at a concentration of about 0.15 mg/L. Surprisingly, the replacement of the promoter ermE*p with the stronger one *gapdh*p (from *E. lenta*) reduced the production of **2**. The effect of promoter strength on the production of AMP in *S. lividans* 66 was quite similar to the production of jadomycin B^50^ and FR-900098^51^ in engineered hosts. The result indicates that the moderate promoters sometimes show the highest titer.

As shown in Fig. 5a, the host strain carrying six genes *alpG-L* produced three other phosphonates with resonance (δ 18.3, 16.2, and 14.0 ppm) corresponding to HEP, HMP, and one unidentified compound. Those three phosphonates were more considerable than that of **2**, suggesting that optimizing the expression strength of six genes is vital for product improvement of **2** in *S. lividans* 66.

**Fig. 6:**
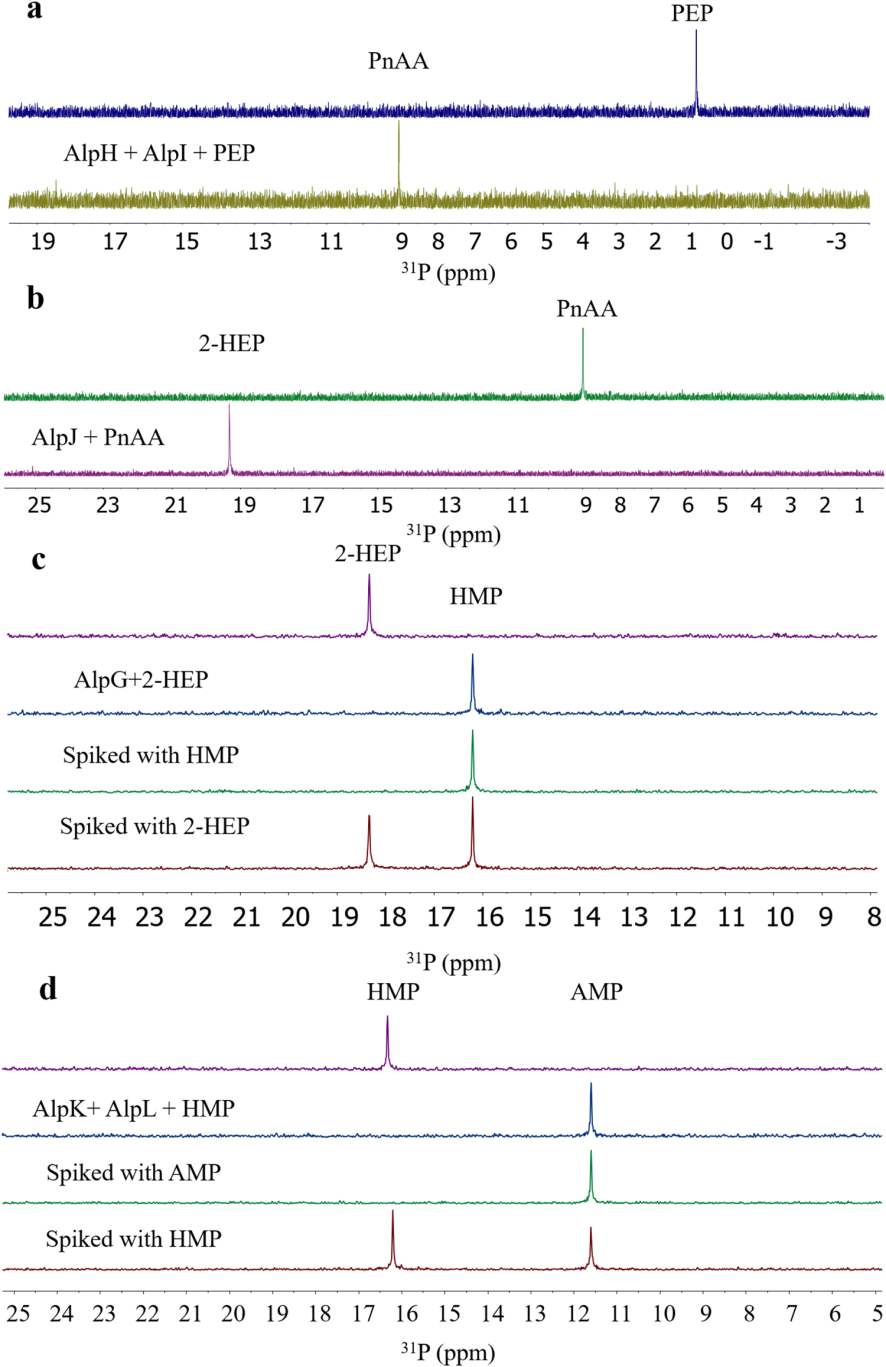
Biochemical characterization of AlpG-L. **a** ^31^P NMR spectrum of the incubation of His_6_-AlpH and His_6_-AlpI with PEP. **b** ^31^P NMR spectrum of the incubation of His_6_-AlpJ with PnAA. **c** ^31^PNMR spectrum of the incubation of His_6_-AlpG with 2-HEP. **d** ^31^P NMR spectrum of the incubation of His_6_-AlpK and His_6_-AlpL with HMP.

To find the bottlenecks in the production of **2** in *S. lividans* 66, we investigated the metabolic profiles of the culture extract when combinations of six genes *alpG-L* were introduced into *S. lividans* 66. Due to the stronger bond dissociation energy of the O-P bond in PEP versus the C-P bond in PnPy, the equilibrium disfavors the formation of C-P bond by a factor of at least 500^52^. Therefore, we had to heterologously express both alpH and I in the host strain. ^31^P NMR analysis showed that the introduction of *alpHI* led to the accumulation of aminoethylphosphonate (AEP), similar to the previous gene *phpC* knockout experiments in the biosynthesis of the herbicide phosphinothricin tripeptide (PTT)^53^. However, we could not find any peak corresponding to PnAA in the ^31^P NMR spectrum and found the trace amount of PnAA in the UPLC-MS, indicating that most of the produced PnAA were converted into 2-AEP by nonspecific aminotransferases in the host strain. To confirm the function of AlpH and I, we overproduced them in *E. coli* BL21(DE3) as a soluble *N*-his_6_-tagged protein. After incubation, the reaction mixture was subjected to IMAC and HILIC chromatography to remove all phosphates and much impurities before ^31^P NMR analysis. In vitro assay showed that PEP was converted to only PnAA by AlpH and AlpI in the presence of thiamine diphosphate and Mg^2+^ (Fig. 6a). The introduction of *alpJ* into *S. lividans* 66 *alpHI* led to the appearance of 2-HEP and a minor compound 2-AEP. However, in vitro assay showed that PnAA was converted to only 2-HEP by AlpJ in the presence of reduced nicotinamide adenine dinucleotide (NADH) (Fig. 6b).

The culture of *S. lividans alpGHIJ* led to the accumulation of HMP in the extract (Fig. 5a). Then we cloned and overexpressed *alpG* in *E. coli* with an N-terminal hexahistidine tag (6 × His-tag) and purified by nickel-nitrilotriacetic acid (Ni^2+^-NTA) affinity chromatography. The conversion of 2-HEP to HMP in the presence of purified protein with Fe(II) and O_2_ was monitored by ^31^P NMR spectroscopy (Fig. 6c). Given the proposed activity of AlpK as a NAD(P)-dependent alcohol dehydrogenase and AlpL as an aminotransferase, we expected to find **2** in the culture broth of the *S. lividans alpGHIJKL* mutant. In vitro assay showed that HMP was converted to only AMP by AlpK and AlpL in the presence of NAD^+^ (or NADP^+^) and l-glutamate (or l-aspartate) (Fig. 6d). When we express the six required genes *alpG-L* in the host strain *S. lividans* 66, a small amount of **2** was produced. However, we found the accumulation of intermediates 2-HEP, HMP, and one unidentified phosphonate with a strong resonance (δ14.0 ppm) in the culture. The result clearly showed that the expression of *alpK* and *alpL* is the bottleneck for the production of **2** (Fig. 5a). To validate the accuracy of the ^31^P NMR technology, we determined their cognate mRNA level using real-time quantitative PCR. As shown in Fig. 5b, all the six genes in the reconstructed gene cluster were substantially transcribed under different promoters, including the original one, ermE*p, and *gapdh*p (*E. lenta*). The first four genes *alpG*, *H*, *I*, and *J*, under the original, ermE*p and *gapdh*p promoters, showed ~5, 60, and 130-fold higher expression levels than the internal control *hrdB*^54^. In contrast, both *alpK* and *L* under the native, ermE*p, and *gapdh*p promoters showed low expression levels. The result confirms that the transcriptional expression of *alpK* and *L* limits the high production of **2** in the host strain.

Furthermore, this gene cluster *alpGHIJKL* contains intricately internal regulation, including embedded feedforward and feedback loops. For example, the expression of *alpK* and *L* has a negative feedback on the biosynthesis of HMP catalyzed by AlpG, H, I, and J. When we expressed *alpG*, *H*, *I*, *J*, *K*, and *L* into *S. lividans* 66, the genes *alpG*, *H*, *I*, and *J* under the ermE*p promoter showed ~30% lower expression levels than those in the host strain transformed with plasmids containing only *alpG*, *H*, *I*, and *J* (Fig. 5b). The host strain produced only HMP without accumulating intermediates when we expressed *alpG*, *H*, *I*, and *J* into *S. lividans* 66 (Fig. 5a). However, the cultures had small amounts of **2** with the accumulation of three intermediates, HMP, 2-HEP, and one unidentified phosphonate with a strong resonance (δ14.0 ppm) when we expressed *alpG*, *H*, *I*, *J*, *K*, and *L* into *S. lividans* 66. The redundancy and complexity of these embedded regulations make it difficult to induce the expression and enhance the production of **2** by only inserting various promoters upstream of this single operon.

### Enhanced production of AMP using a promoter-insulator-RBS strategy

**Fig. 7:**
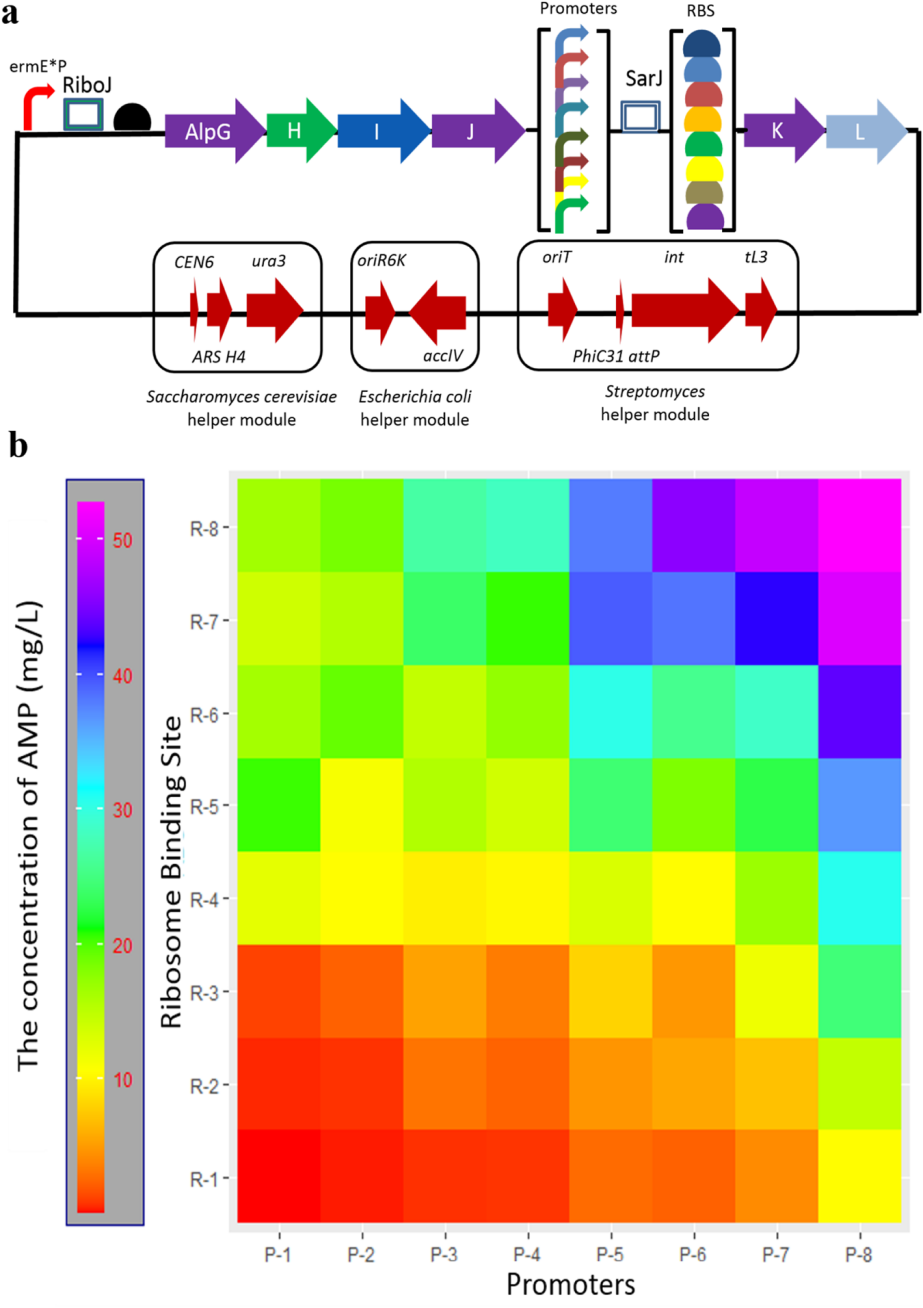
Production improvement of AMP in *S.lividans* using a promoter-insulator-RBS strategy. **a** A scheme of the combined eight promoters and eight RBSs to form a full combinatorial library ofexpression control elements with the insulator RiboJ. **b** Heat maps show the production of AMP for all combinations of different promoters (P, columns) and RBSs (R, row). Each value was obtained bymeasuring AMP concentration with three experimental duplications. Source data are provided as a Source Data file.

Engineering microorganisms for high production of **2** requires metabolism reprogramming to ensure high flux toward **2**. Based on the analysis above, we applied a global bottom-up strategy to systematically break the original regulations and replace them with well-characterized genetic parts and circuits to highly induce the expression of alpGHIJKL and enhance the production of **2**. According to the transcriptional expression levels, the six genes were divided into two operons, *alpGHIJ*, and *alpKL*. Each operon was controlled and regulated with synthetic promoters and RBS. To confirm the applicability of insulators for eliminating the negative feedback, we designed promoter-insulator-RBS cassettes (ermE*p-RiboJ-RBS and *gapdh*p-SarJ-RBS, Supplementary Tables 5–7 and Supplementary Fig. 10) upstream of each operon (*alpGHIJ* and *alpKL*) (Fig. 7a). Interestingly, the transformed strain produced the major phosphonate **2** and the minor HMP without accumulating other intermediates (Fig. 5a).

Furthermore, the first four genes *alpG*, *H*, *I*, and *J* showed similar expression levels as those in the strain transformed with *alpGHIJ*, much higher than those in the strain transformed with *alpGHIJKL*. The two genes *alpKL* exhibited much higher transcriptional levels than those in the strain transformed with *alpGHIJKL*. All the above data confirm the capability of the insulators RiboJ and SarJ to eliminate the interference and negative feedback between two operons.

To optimize the regulation system, we constructed promoter and RBS libraries that contain eight well-characterized strong promoters and RBSs (Supplementary Tables 5, 6) with different strengths to highly induce *alpKL*^14,31,32,54,55^. Furthermore, we introduced the well-elucidated 79-nt SarJ insulator between the pairwise combined eight promoters and eight RBSs to improve the predictability of the combination, resulting in 64 promoter-insulator–RBS regulatory cassettes upstream of *alpKL* (Fig. 7b). The maximum production of **2** reached 52.6 mg/L, ~500 times higher than that in the wild-type strain when *gapdh*p (*Rhodococcus erythropolis*)-SarJ-Helicase (φC31) (P8R8RC) was used. Furthermore, the culture accumulated only **2** without intermediates and shunt products (Fig. 5a), indicating the high applicability and designability of the bottom-up promoter-insulator-RBS strategy for enhancing the production of final products of interest.

### Chemical synthesis

**Fig. 8:**
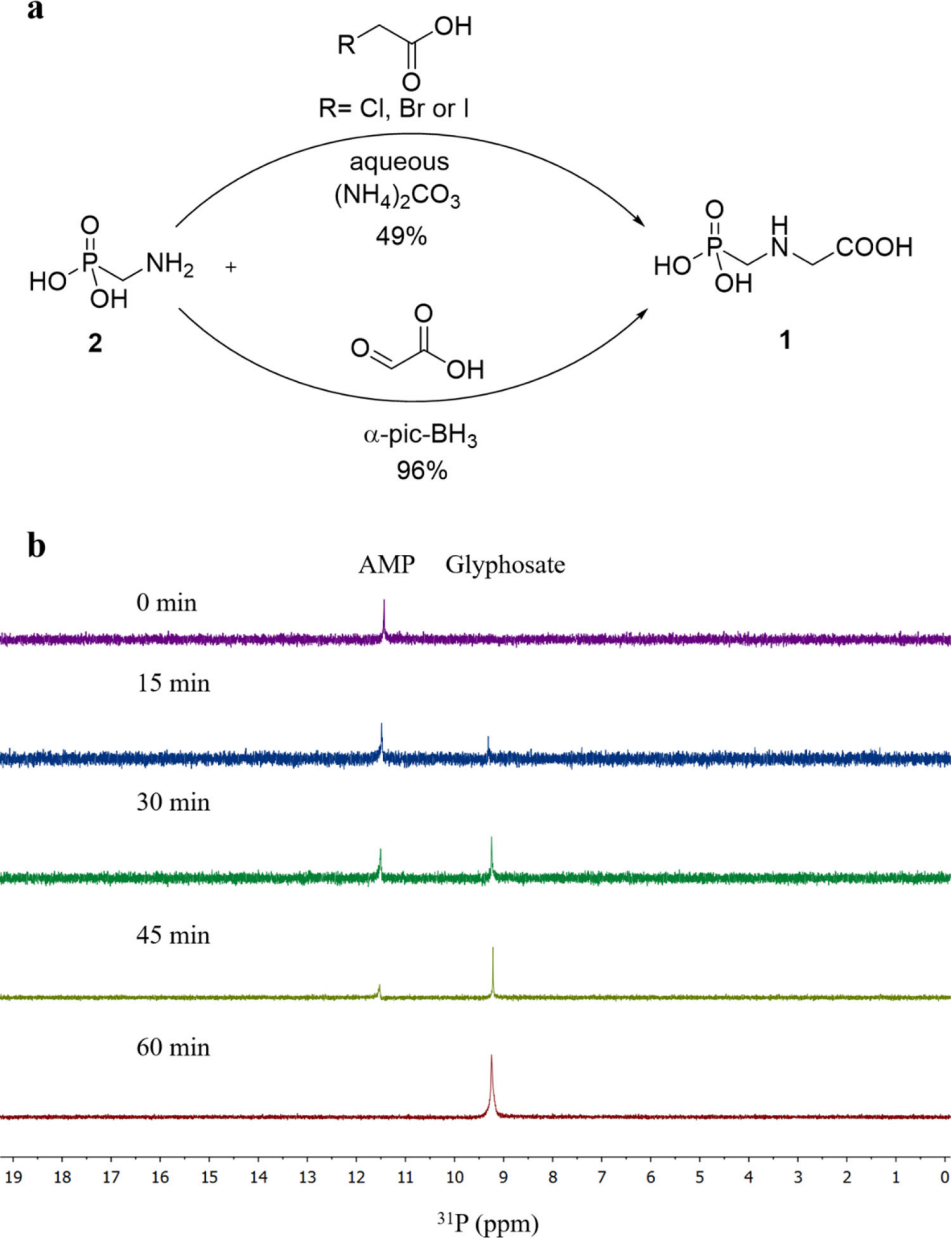
Chemical conversion of 1 from 2. **a** Semisynthesis of **1** from microbially produced **2**. **b** ^31^P NMR traces of reaction product of **2** with glyoxylic acid and α-pic-BH_3_ (1 mmol) after incubation for 60 min at room temperature in H_2_O-AcOH (5:1) solution.

With **2** on hand, we sought to synthesize glyphosate from **2** chemically. As the primary amine is more nucleophilic than the alcohol, we tried to find a one-pot synthesis of glyphosate from **2** in aqueous conditions without protecting the phosphonic acid groups. As halide ions are excellent leaving groups in the SN_2_ reactions, we initially tried to use haloacetic acids as reactants to react with **2** to generate glyphosate (Fig. 8a and Table 2). In this approach, we had to control a 1: 1 ratio of **2** to haloacetic acids to prevent the formation of some higher *N*-alkylated products.

**Table 2:** SN_2_ reaction of AMP (2) with haloacetic acids toward glyphosate (1).

Entry	R	Temperature (°C)	Time (h)	Yield (%)
1	-Cl	50	1	2.5
2	-Cl	50	2	3.9
3	-Cl	50	3	4.6
4	-Cl	90	1	13.0
5	-Cl	90	2	11.9
6	-Cl	90	3	12.8
7	-Br	50	1	13.7
8	-Br	50	2	14.0
9	-Br	50	3	15.2
10	-Br	90	1	30.4
11	-Br	90	2	30.2
12	-Br	90	3	30.7
13	-I	50	1	5.8
14	-I	50	2	25.3
15	-I	50	3	34.6
16	-I	90	1	49.2
17	-I	90	2	49.2
18	-I	90	3	49.1

The conversion of haloacetic acids and **2** to glyphosate was positively influenced by the type of halogen atom, reaction temperature, and reaction duration. Different mono-haloacetic acids during SN_2_ reaction with **2** were assessed and analyzed. Idioacetic acid showed a significantly higher conversion than bromoacetic acid and chloroacetic acid. The possible explanation is the decreasing order of the carbon-halogen bond strength as C-Cl, C-Br, and C-I bond strengths are 338, 276, and 238 kJ/mol, respectively^56^. The conversion of **2** and haloacetic acids to glyphosate rose with increasing reaction temperature and time. The higher yield was achieved for all three haloacetic acids at 90 °C than 50 °C. After 60 min, the maximum glyphosate yields of ~13, 31, and 49% were obtained when three haloacetic acids were reacted with **2**, respectively at 90 °C (Table 2, entries 4, 10, and 16).

The second strategy to synthesize glyphosate is the reductive amination of aldehydes. Generally, the approach involves the generation of an imine or iminium intermediate followed by reduction to an alkylated amine. Two distinct approaches are generally adopted for reductive amination reactions: indirect and direct. The former involves the reduction of an imine, which is generated from an amine and a carbonyl reactant by nucleophilic addition and subsequent dehydration. The latter involves mixing two reactants and a suitable hydride source, which selectively reduces the in situ generated imine. The direct method is perhaps the most straightforward and is therefore widely practiced^57^. A wide range of reducing agents have been extensively explored and used in the process of reductive amination. These include sodium NaBH_4_^58^, NaBH_3_CN^59^, NaBH(OAc)_3_^60^, SnCl_2_^61^, α-pic-BH_3_^62^, [RuCl_2_(*p*-cymene)]_2_/Ph_2_SiH_2_^63^, and B_10_H_14_^64^.

In our case, **2** is dissolved in only water and insoluble in organic solvents. Furthermore, water is a nontoxic, nonflammable, and environmental-friendly solvent. Therefore, we conducted the reductive amination in water to synthesize glyphosate. Among all reducing agents used, α-pic-BH_3_ is a very stable solid that can be stored without decomposition. Therefore, α-pic-BH_3_ is an excellent alternative for reductive aminations in water, methanol, or even under solvent-free conditions.

We accomplished the reductive amination of **2** with glyoxylic acid using α-pic-BH_3_ in H_2_O and AcOH at room temperature for 1 h (Fig. 8). Finally, glyphosate was obtained in a high yield (96%, Supplementary Figs. 12, 13). The same reaction was conducted on a larger scale (1 g), and glyphosate was obtained in a similar yield.

After we studied the biosynthesis of the antibiotics argolaphos, we elucidated the full AMP biosynthetic pathway. We advanced a semisynthesis process to produce the herbicide glyphosate by fermentation of engineered *S. lividans* to produce **2**, followed by chemical conversion to glyphosate. Using synthetic biology approaches, we engineered a metabolic pathway in *S. lividans* 66 to yield a key intermediate **2** at high production. Furthermore, we developed a practical, efficient, and scalable chemical process for converting **2** to glyphosate using one-step reductive amination at room temperature. These critical developments in *Streptomyces* strain engineering, fermentation, and glyphosate synthetic chemistry set the stage for an industrial and green process capable of meeting the world demand for glyphosate from an alternative source independent of the severe pollutions associated with current chemical synthesis.

## Methods

### Materials and reagents

All reagents, kits, and chemicals used for molecular biology and chemistry experiments were obtained from commercial sources, including Sigma-Aldrich Shanghai Trading Co Ltd. (Shanghai, China), Thermo Fisher Scientific (Waltham, MA, USA), New England Biolabs, and used without further purification unless otherwise specified. Enzymes were purchased from Takara Biotechnology (Dalian, China) or Vazyme Biotech (Nanjing, China) unless otherwise specified.

All plasmids and strains used in this study are listed in Supplementary Table 1. The composition of culture media used in this study is listed in Supplementary Table 8. *Streptomyces lividans* 66 and *S. monomycin* NRRL B-24309 were obtained from the Agricultural Research Service Culture Collection (Peoria, IL, USA). *E. coli* BW25141 and WM6026 were provided by Dr. William Metcalf (University of Illinois, Urbana, IL, USA). *S. cerevisiae* HZ848 and the plasmid pRS416 were provided by Dr. Huimin Zhao (University of Illinois, Urbana, IL, USA). The plasmid pET-15b was provided by Dr. Huan Wang (Nanjing University, Nanjing, China). All promoters, RBS, primers, and oligonucleotides were synthesized by Tsingke biological technology (Fuzhou, China).

### Instrumentation

^1^H NMR spectra were measured on a JEOL-ECS-400 (400 MHz) spectrometer. Chemical shifts are reported in ppm from the solvent resonance or tetramethylsilane (TMS) as the internal standard. Splitting patterns were recorded as follows: *s* = singlet, *d* = doublet, *t* = triplet, *m* = multiplet, *br* = broad peak. ^13^C NMR spectra were recorded on a JEOL-ECS-400 (100 MHz) spectrometer with complete proton decoupling. Chemical shifts were reported in ppm from the solvent resonance as the internal standard. ^31^P NMR spectra were recorded on a JEOL-ECS-400 (162 MHz) spectrometer. Chemical shifts were recorded in ppm from 85% H_3_PO_4_ (0 ppm) resonance as the external standard. The spectra were analyzed using the Delta NMR software or MestRenova software version 8.1.1. The LC/MS system used was a Waters^®^ Xevo^®^ G2-XS Qtof high-resolution mass spectrometer coupled with an ACQUITY^®^ UPLC^®^ I-Class Bio System. Data were collected in positive or negative ion mode using a SONAR quadrupole window, which provides a full scan MS over the TOF mass range of 100–1000 Da. The data was processed post-acquisition in UNIFI. HPLC measurements were performed on a 7900HT Essentia Pre LC-16P (Essentia, Kyoto, Japan) equipped with a UV detector (SPD-16). PCR was performed on a Bio-Rad T100^TM^ Thermal Cycler using Phusion^®^ or Q5^®^ High-Fidelity DNA polymerase. Real-time PCR was performed using an Applied Biosystems 7900HT Fast Real-Time PCR System. High-speed refrigerated centrifuge Avanti was performed using an American Beckman Coulter Co., Ltd J-26S XP. The pH measurements were performed on a PHS-3B digital pH meter (Shanghai Hongyi Instrument Co. Ltd.).

### Whole-genome sequencing and analysis

Genomic DNA of the strain *S. monomycin* NRRL 24309 was extracted with the SDS method^65^. The harvested DNA was detected by the agarose gel electrophoresis and quantified by Qubit^®^ 2.0 Fluorometer (Thermo Scientific). A total amount of 1 μg DNA per sample was used as input material for the DNA sample preparations. Sequencing libraries were generated using NEBNext^®^ Ultra™ DNA Library Prep Kit for Illumina (NEB, USA) following the manufacturer’s recommendations, and index codes were added to attribute sequences to each sample. Briefly, the DNA sample was fragmented by sonication to a size of 350 bp. DNA fragments were end-polished, A-tailed, and ligated with the full-length adapter for Illumina sequencing with further PCR amplification.

At last, PCR products were purified (AMPure XP system), and libraries were analyzed for size distribution by Agilent2100 Bioanalyzer and quantified using real-time PCR. The whole genome was sequenced using Illumina NovaSeq PE150 at the Beijing Novogene Bioinformatics Technology Co., Ltd. Illumina PCR adapter reads, and low-quality reads from the paired-end were filtered by the step of quality control using our own compiling pipeline. All good quality paired reads were assembled using the SOAP denovo^66^, SPAdes^67^, and ABySS^68^ into a number of scaffolds. Then the filter reads were handled by the next step of the gap-closing. The assembled genomic data for each strain in fastq format were uploaded to Rapid Annotation Using Subsystem Technology (RAST) server for annotation^28^. The annotated genomes were analyzed using RAST tools and antiSMASH^29^.

### Streptomycete cultivation and expression analysis

Spores from *S. lividans* 66 transformed with different combinations of *alp* genes and promoters were inoculated into MYG medium (Supplementary Table 8) at 28 °C with constant shaking (200 r.p.m.). After 72-h cultivation, the total RNA was isolated with a TRIzol reagent (Invitrogen, USA) according to the manufacturer’s instructions. The cDNA was performed using M-MLV First-strand cDNA Synthesis Kit (Invitrogen, USA). Real-time PCR was performed with SYBR Green PCR Master Mix (Bio-Rad). Primers were designed using the online tool provided by Integrated DNA Technologies (https://www.idtdna.com/scitools/Applications/RealTimePCR/). The reaction system was generated by gently mixing 10 µL of 2 × SYBR Green Mix, 1 µL of cDNA templates, 1 µL of each primer at a concentration of 10 pmol µL^−1^, and 7 µL of *dd*H_2_O in each well of the 96-well plate. Amplification was carried out using the following program: 3 min at 50 °C, 3 min at 95 °C for one cycle; 20 s at 95 °C, 30 s at 60 °C, and 30 s at 72 °C for 30 cycles with a final cycle of 10 min. The endogenous gene *hrdB*, encoding RNA polymerase sigma factor, was employed as the internal control for all real-time RT-qPCR experiments. The transcriptional levels of all target genes were normalized against the expression level of the reference gene.

### DNA manipulation and gene cluster reconstruction

Gene cluster fragments (each 2000–3000 bp) were PCR-amplified from the genomic DNA isolated from *S. monomycin* NRRL B-24309 using an UltraClean Microbial DNA Isolation Kit (Qiagen). All primer sequences were summarized and listed in Supplementary Data 1. The *S. cerevisiae* helper fragment was PCR-amplified from the plasmid pRS416, whereas the *E. coli* helper fragment and the *S. lividans* helper fragment were PCR-amplified from the plasmid pAE4. Following electrophoresis, all PCR products were individually gel-purified from 0.7% agarose using Qiagen Gel Purification Kit. Each gene expression cassette, including gene, promoter, insulator, or RBS, was assembled by overlap extension PCR (OE-PCR). To ensure yeast homologous recombination’s high efficiency, the cassette was designed to generate a ~200 bp overlap region with adjacent fragments. Individual cassettes (2–3 kb, 300 ng each) were gently mixed and precipitated with 100% ethanol. After air drying, the resulting DNA pellet was resuspended in 4 µL of deionized water and stored at −20 °C for yeast transformation.

### Yeast transformation

The concentrated mixture of DNA fragments was electroporated into the yeast *S. cerevisiae* HZ848. An aliquot of 0.5 mL of *S. cerevisiae* HZ848 cells was inoculated into a 50 mL YPAD liquid medium (Supplementary Table 8), and the culture was shaken at 250 rpm and 30 °C for about 5 h until the OD_600_ value reached 1.0. Yeast cells were collected by high-speed centrifugation (5000 × *g*) at 4 °C for 5 min. The supernatant was immediately removed, and the cell pellet was washed with 50 mL ice-cold deionized water, followed by washing with ice-cold 1 M sorbitol and finally resuspended in 250 mL ice-cold sorbitol. An aliquot of 50 µL of yeast cells together with a 4 µL DNA mixture was electroporated in a 0.2-cm chilled electroporation cuvette at 1.5 kV with a time constant of 5.0–5.2 ms, followed by immediate addition of 1 mL pre-warmed (30 °C) YPAD medium to respend the transformed cells. The cells were shaken at 30 °C and 250 rpm for 1 h. The transformed cells were then collected by high-speed centrifugation, washed with 1 M sorbitol several times to remove the YPAD medium, and finally resuspended in 1 ml sorbitol at room temperature. Aliquots of 50–100 µL were spread on SC-Ura plates, and the plates were incubated at 30 °C for 2–4 days until colonies appeared^69^.

### Restriction digestion analysis

Several colonies were randomly picked to SC-Ura liquid media and grown at 30 °C and 250 rpm for one day, after which the plasmids were extracted from yeast cells using the TIANprep Yeast Plasmid DNA Kit (TIANGEN, Beijing, China). An aliquot of 50 µL of *E. coli* strain BW25141 together with a 2 µL isolated plasmid was electroporated in a 0.2-cm chilled electroporation cuvette at 2.5 kV with a time constant of 5.0–5.2 ms, followed by immediate addition of 1 mL pre-warmed (37 °C) SOC medium to respend the transformed cells. The cells were shaken at 37 °C and 250 rpm for 1 h. The cells were spun down, and 800 mL of SOC medium was removed. The cell pellet was resuspended with the remaining 200 mL of SOC medium, and the cells were spread on LB plates supplemented with 50 µg mL^−1^ apramycin (Apr). The plates were incubated at 37 °C for about 18 h until colonies appeared. Colonies were inoculated into 5 mL of LB media supplemented with 50 µg mL^−1^ apramycin and grew at 37 °C for 12–16 h. *E. coli* plasmids isolated from the liquid culture were then verified through at least two separate restriction digests for each plasmid. Usually, appropriate restrictive enzymes were chosen to cut the target plasmid at multiple sites that will result in multiple fragments with obviously various sizes. The reaction mixtures were loaded to 1% agarose gels to check for the correct restriction digestion pattern by DNA electrophoresis.

### Conjugation and heterologous expression in *S. lividans*

The verified plasmids were electroporated to an auxotrophic strain *E. coli* WM6026 and selected on LB agar plates supplemented with 2, 6-diaminopimelic acid (DAP) and apramycin. These transformants were then used as the donors for conjugative transfer of the assembled plasmids to *S. lividans* 66^70^. The verified plasmid was mixed with *E. coli* WM6026 cells, and the mixture was put into a chilled electroporation cuvette. The cells were electroporated at 2.5 kV and quickly resuspended by adding SOC medium and DAP. Then the transformed cells were cultured in a shaker at 37 °C, 250 rpm for 1 h. The culture was spread on an LB-Apr^+^-DAP plate, and the plates were incubated at 37 °C for 16 h until colonies appear. A single colony was inoculated from each plate into 2 mL of LB supplemented with 50 µg mL^−1^ apramycin and 10 mL of 38 mg mL^−1^ DAP and grew at 37 °C until OD_600_ reaches 0.6–0.8. The cell culture was centrifugated in an Eppendorf tube, and the cell pellet was washed with the fresh LB medium twice. The cell pellets were resuspended with the fresh LB. The resuspended cells were mixed with *S. lividans* spores by pipetting and spot 2 mL aliquots onto R2 no-sucrose plates. After all the spotted drops were absorbed entirely into the R2 no-sucrose plates, the plates were incubated at 30 °C for about 18 h. The plates were flooded with a mixture of nalidixic acid and apramycin at a 1 mg/mL concentration. The plates were incubated at 30 °C for about 3–5 days until exconjugants appeared. A single exconjugant was picked and restreaked on ISP2 plates supplemented with 50 µg mL^−1^ apramycin and grew for about 3–5 days. A single colony was inoculated into the ATCC172 liquid medium supplemented with 50 µg/mL apramycin in a tube for 3 days and then inoculated into 100 mL ISP2 liquid medium supplemented with 50 µg/mL apramycin. The cells were incubated for seven days at 30 °C and 250 rpm.

### Expression and purification of AlpH, I, J, G, K, and L

Linear pET-15b was prepared by cutting the circular plasmid with NdeI and PCR-amplifying the resulting product. The PCR reaction (50 μL) contained 1X Fail-Safe buffer G, 50 μM of each primer, 20–40 ng of linear plasmid, and 0.05 U µL^−1^ Q5 High-Fidelity DNA polymerase. The line plasmid was amplified through the following program on a thermocycler: 98 °C 3 min, 30 cycles of 98 °C 10 s, 58 °C 1 min, 72 °C 5 min, then 72 °C 8 min. The PCR product was digested with the restriction enzyme DpnI and purified with the Wizard^®^ SV kit (Promega).

pET-15b-AlpG (or H, I, J, K, and L) was prepared by amplifying the gene from *S. monomycin* NRRL B-24309 genomic DNA. The 50 μL reaction contained 1X Fail-Safe buffer G, 1 μM of each primer (Supplementary Data 1), 250 ng gDNA, and 0.05 U µL^−1^ Q5 High-Fidelity DNA polymerase. The system was run through the following program on a thermocycler: 98 °C 2 min, 25 cycles of 98 °C 15 s, 58 °C 15 s, 72 °C 1 min, then 72 °C 8 min.

The PCR samples were run on a 1% agarose gel and purified using the Wizard^®^ SV kit (Promega). The products (200 ng) were used directly for Gibson assembly with 100 ng linear pET-15b DNA. The 20 μL ligation reactions contained a 1X NEB Gibson master mix. The ligation reactions were incubated at 50 °C for 1 h.

An aliquot (~2 μL) was used to transform chemically competent *E. coli* DH5α cells. The transformed cells recovered in SOC media for one hour at 37 °C before being plated on LB plates supplemented with ampicillin and grown overnight at 37 °C. Colonies were selected, and the cells were grown overnight at 37 °C. The plasmid was extracted, and the sequence was confirmed by sequencing at Boshang Biotech Company (Fuzhou, China).

The pET-15b expression vectors were chemically transformed into *E. coli* BL21 Rosetta (DE3) cells. The transformed cells were grown in LB media supplemented with ampicillin (100 μg mL^−1^) and chloramphenicol (25 μg mL^−1^) at 37 °C until the OD_600_ value reached 0.6. The temperature was then adjusted to 18 °C, and the expression was induced by the addition of 0.3 mM IPTG. After 14–20 h, the transformed cells were collected by centrifugation (12,000 × *g*, 10 min, 4 °C). Cells were flash-frozen in liquid nitrogen and stored at −80 °C. Cells harvested from 1000 mL of LB were resuspended in 25 mL of buffer C (50 mM HEPES pH 7.5, 200 mM KCl, 10% (v/v) glycerol) supplemented with 20 mM imidazole, 0.4 U mL^−1^ DNase, and 1 mg mL^−1^ lysozyme at room temperature. Cells were lysed by sonication (3 × 45 s with 10 min rocking periods at 4 °C, and the insoluble debris was removed via centrifugation (35,000 × *g*, 1 h, 4 °C).

Clarified lysates were affinity purified using a 5 mL nickel-nitrilotriacetic acid (Ni-NTA) column previously equilibrated with buffer C supplemented with 20 mM imidazole. Protein was eluted with buffer C supplemented with 250 mM imidazole. Elution fractions were combined and concentrated using a 30 kDa MWCO centrifugal filter and then buffer exchanged into buffer C with either a NAP-25 column or dialysis tubing following the manufacturer’s instructions. Protein was flash-frozen in liquid N_2_ and stored at −80 °C for future use.

### AlpH and AlpI coupling enzymatic reaction

The reaction mixture (500 µL) contained 1.5 mM thiamine diphosphate, 2 mM Mg^2+^, 10 µM AlpH, 10 µM AlpI, and 2 mM PEP in 50 mM HEPES buffer (pH 7.5). The reaction was initiated by the addition of 10 µM AlpI. The reaction mixture was incubated at 28 °C for 5 h, subjected to IMAC and HILIC chromatography, and then analyzed by ^31^P NMR spectroscopy.

### AlpJ enzymatic assay

The biochemical reaction was performed in 50 mM HEPES pH 7.25 with 20 µM AlpJ, 2 mM PnAA, 5 mM NADH, and 5% glycerol in a total volume of 500 µL. The reaction mixture was incubated at 28 °C for 16 h, subjected to IMAC and HILIC chromatography, and then analyzed by ^31^P NMR spectroscopy.

### AlpG enzymatic assay

The reaction mixture (500 µL) contained 10 µM AlpG, and 2 mM 2-HEP in 50 mM HEPES buffer (pH 7.5). The reaction mixture was incubated at 28 °C for 2 h, subjected to IMAC and HILIC chromatography, and then analyzed by ^31^P NMR spectroscopy.

### AlpK and AlpL coupling enzymatic assay

The reaction mixture (500 µL) contained 2 mM Mg^2+^, 1 mM NAD^+^, 1 mM NADP^+^, 5 mM l-glutamate or l-aspartate, 2 mM PLP, 10 µM AlpK, 10 µM AlpL, and 2 mM HMP in 50 mM HEPES buffer (pH 7.5). The reaction was initiated by the addition of 10 µM AlpK. The reaction mixture was incubated at 28 °C for 1 h, subjected to IMAC and HILIC chromatography, and then analyzed by ^31^P NMR spectroscopy.

### Phylogenetic analysis

A multiple sequence alignment of six discrete MPnS-related proteins was generated using the Clustal Omega^71^ web tool (http://www.ebi.ac.uk/Tools/msa/clustalo/) with the default parameters. All phylogenetic analysis was performed using the Molecular Evolutionary Genetics Analysis (MEGA) X^72^. Maximum likelihood phylogenetic trees were created in MEGA using the standard parameters. A WebLogo frequency plot was generated from a Clustal Omega alignment of all of the sequences using the standard parameters^73^.

### NMR analysis

The liquid culture was centrifuged, and the pellets were removed immediately. The liquids were evaporated to dryness using a rotary evaporator. The crude sample was prepared by dissolving 1.0–3.0 mg in 250 µL of D_2_O (Sigma-Aldrich, 99.96% atom%D). NMR spectra were recorded on a JNMR ECZ-400s 400 MHz spectrometer equipped with a 5 mm FG/TH auto-tune probe. Samples were run at 25 °C during acquisition. Standard pulse sequences were set up for each experiment, including ^1^H, ^13^C, ^31^P, and ^1^H-^31^P HMBC. Spectra were recorded with the Delta NMR software and analyzed with the software MestReNova 8.1.1.

### LC-MS analysis

The crude sample was redissolved in H_2_O and then sonicated at 0 °C for 5 min. The sample was injected into the system for UPLC–HRMS detection (Waters) with a HILIC column (2.1 × 50 mm 1.7 μm). The gradient was: 0–5 min, 100% B; 5–15 min, 100% B–40% B, 15–20 min, 40% B–100% B; 20–35 min 100% B at a flow rate of 0.2 Ml min^−1^. Solvent A: 10 mM NH_4_HCO_3_; Solvent B: 10 mM NH_4_HCO_3_, 90% Acetonitrile (ACN). Samples were analyzed by precursor ion screening in negative mode (monitoring m/z 50).

### Isolation and purification of argolaphos A (3) from *S. monomycini* B-24309

*Streptomyces m*. B-24309 was cultivated in 250 mL of ATCC172 seed medium for 3 days at 30 °C on a platform shaker rotating at 200 rpm before inoculation of 20 L of solid ISP4 plates. After incubating at 30 °C for 10 days, the agar-solidified medium was liquefied by repeated freezing and subsequent thawing. Argolaphos were isolated and purified using the modified version of the previous procedure^15^. Briefly, The resulting supernatant (15 L) was generated by filtration on a Büchner funnel before extensive concentration via rotary evaporation. A total volume of 2 l of 90% methanol was added and shaken vigorously by hand. The supernatant was filtered and concentrated to dryness and the residue dissolved in 50 mL of 0.1% aqueous acetic acid, which was applied to a 40 mL Fe(III) IMAC column. The phosphonates were eluted from the column with 100 mM aqueous NH_4_HCO_3_. Several rounds of ^31^P NMR guided size-exclusion chromatography were used to isolate and purify the phosphonates. The elute chromatographed over different glass Sephadex LH-20 columns (from 40 mm × 1400 mm to 10 mm × 1800 mm gel bed) eluted with distilled H_2_O to produce many fractions. Based on ^31^P NMR analysis of each fraction, fractions containing phosphonates were combined and dried with a rotary evaporator to give the sample, which was dissolved in 0.5 mL of distilled H_2_O and further purified using a 10 mm × 250 mm HILIC column (Atlantis^®^HILIC Silica, 5 µm) and gradient elution, yielding compounds **3** (3 mg) and **4** (1 mg).

### Reduction of argolaphos A (3) to compound 8

Indium-mediated reduction of **3** to the corresponding amine **8** was accomplished using the previous method^26^. Briefly, compound **3** (0.5 µmol) was put into a 2:1 solution of ethanol and saturated aqueous. NH_4_Cl (1 mL) in a 5 mL round bottom flask equipped with a Claisen condenser and a magnetic stirring bar. An appropriate amount of In powder (2 equiv.) was added, and the system was heated to reflux for about 8 h. After the reaction monitored by HILIC HPLC was complete, the mixture was cooled down, filtered over Celite, concentrated, and dissolved in distilled H_2_O. Furthermore, the product was purified using a 10 mm × 250 mm HILIC column (Altantis^®^ HILIC Silica, 5 µm) and a gradient elution, yielding the compound 8 (95% yield). The gradient used was: 20 min at 100% solvent B (0.1 formic acids in ACN) then a linear gradient to 60% solvent A (0.1% formic acids in distilled H_2_O) over 40 min. The flow rate was 3 mL min^−1^. The retention time was 23 min.

### Marfey’s analysis

A dried sample of ~0.1 mg of the peptide was dissolved in 2 mL of 6 N HCl in an ampule and heated to reflux for 14 h in an oil bath pot^74^. The hydrolysate was dried entirely under N_2_ in a glass vial. 3.6 μmol of a 1% acetone solution of FDAA (*N*-(5-fluoro-2,4-dinitrophenyl)-d-alaninamide) and 20 μmol of 1 M NaHCO_3_ were added to the peptide to initiate the derivatization reaction in the vial. The reaction mixture was then heated with an HH-S digital thermostat water bath (Jiangsu Jintan Medical Instrument Factory) at 40 °C for 50 min under agitation and cooled to room temperature. Then, the reaction was quenched by adding 20 μmol of 1 M HCl and then 1 mL of MeOH. The mixture without isolation was analyzed with a Waters^®^ Xevo^®^ G2-XS Qtof high-resolution mass spectrometer coupled with an ACQUITY^®^ UPLC^®^ I-Class BioSystem using a reverse-phase C18 column, and retention times were compared with those of the corresponding amino acid standards. The eluent A comprises 0.05% HCOOH in ACN and eluent B as the water was used through a linear gradient elution mode (eluent A, 10–60%, 60 min) at a flow rate of 0.4 mL min^−1^.

Absolute configuration was determined by comparing the retention time of derivatives from commercially available amino acids and spiked experiments with standard amino acids. Therefore, the absolute configuration of valine and *N*^5^-hydroxyl arginine in compound **3** was determined to be L.

### Quantification of AMP (2) in the culture

Quantification of **2** in this study was based on the previous methods with minor modifications^75,76^. Briefly, samples (1 mL) for **2** measurement were taken after 5-day bacterial culture and was centrifuged (10,000 rpm, 5 min, 4 °C). The pellets were discarded, and the supernatant was then stored at −80 °C for further use. Extraction of **2** was carried out by adding 1 mL of CH_3_OH: CH_3_CN: H_2_O (2:3:1) to 0.1 mL of the samples. The samples were subjected to sonication for 5 min and high-speed centrifugation at 12,000 rpm for 8 min at 4 °C. The pellets were discarded, and the supernatant was treated in a high vacuum centrifuge. The dried samples were dissolved in 50 µL of distilled water and filtered for UPLC/MS analysis. The standard procedure was applied for all the above samples. Dense samples with a higher concentration than the maximum limit of quantitation have to be diluted with the appropriate amount of distilled water. Five repeat injections were used as technical replicates to validate the UPLC/MS method used in this experiment. Three replicates were used to detect the robustness of the AMP extraction method used. From each culture, three samples were extracted, and each sample was analyzed twice.

For the **2** analysis using UPLC/MS, 10 µL of the sample was injected into an ACQUITY BEH amide 1.7 µm column (2.1 × 50 mm; Waters, USA). The autosampler was run at 12 °C, and the column oven was kept at 28 °C. The following solvents were designed for the LC analysis. Solvent A: 66% H_2_O, 33% CH_3_CN, 10 mM CH_3_COONH_4_, 0.04% NH_4_OH, pH 9; solvent B: 10% H_2_O, 90% CH_3_CN, 10 mM CH_3_COONH_4_, 0.04% NH_4_OH, pH 9. The LC program was carried out at 0.4 mL min^−1^, and the injection volume was kept at 10 μL. The program started from 0% B for 2 min, followed by a gradient from 0% B to 100% B within 10 min. Then 100% B was kept for 2 min, followed by a constant balance at 0% B for 5 min. The identification and detection of **2** were achieved by the Agilent UPLC-ESI-QTOF-MS system in positive mode. HRMS acquiring parameters were set below: capillary voltage (3 kV); drying gas temperature (300 °C); drying gas flow rate (12 L min^−1^); nebulizer pressure (35 psi). The scan source parameters were set below: skimmer voltage (65 V), fragmentor voltage (175 V), and octupole RF peak voltage (700 V). Tandem MS acquisition of daughter molecular ion for **2** was treated with fixed collision energy 30 eV in the positive mode. All standard curves were produced using linear regression for the **2** quantifications. The linearity was calculated using nine concentrations, 2, 4, 8, 16, 32, 64, 128, 256, 512 ng mL^−1^ (*n* = 5). The signal-to-noise (SN) ratio required for the limit of detection (LOD) and limit of quantification (LOQ) was built using the SN script conducted in Analyst 1.6.2 software. A time window of 0.5 min prior to the peak of AMP was chosen as noise, and the peak of AMP itself was defined as the signal in a time window of 0.1 min. Both LOD and LOQ were calculated by the lowest concentration of the spiked sample with an SN ratio of at least 3 and 10, respectively. The accuracy and precision were estimated at different concentration levels in each matrix. A recovery of 70–120% is acceptable in this study.

### Chemical synthesis of 1 via a biomolecular nucleophilic substitution (S_N_2) reaction

AMP (**2**) (1 mmol) and haloacetic acids (1 mmol) were added to 0.5 M NaHCO_3_ solution and stirred at 50 °C (or 90 °C) for 3 h.

### Chemical synthesis of 1 by reductive amination

To AMP (**2**) (1 mmol) and glyoxylic acid (1 mmol) in MeOH-AcOH (10:1, 5 mL) was added α-picoline-borane (1 mmol), and the mixture was run for 1 h under agitation at room temperature^62^. The reaction was monitored by ^31^P NMR.

### Purification of phosphonates

In our study, the isolation and purification of phosphonates include four basic steps: 70–100% MeOH precipitation, IMAC chromatography, Sephadex LH-20, and HILIC HPLC with minor modifications according to the property and the concentration of target compounds. Here we provide a brief description of the whole procedure.

After the culture or the reaction was completed, distilled H_2_O was immediately added, and the solution was partitioned with EtOAc twice. The organic layer was discarded. The aqueous fraction was concentrated by rotary evaporation, and an appropriate amount of methanol (MeOH) was added to reach a final concentration of 70–100% MeOH. The pellet was removed by high-speed centrifugation, and the supernatant was dried using rotary evaporation. The step can be repeated several times to remove the highly polar components as much as possible.

The supernatant above was dried using a vacuum centrifuge, and the pellet was dissolved in a 0.1% acetic acid solution and subjected to a Fe(III) IMAC gravity column. The IMAC-packed column was equilibrated entirely with 0.1% aqueous acetic acid. The column was then washed successively with 5 volumes of 0.1% aqueous acetic acid after loading the sample onto the column. The phosphonate materials were easily eluted from the iron IMAC column with 200 mM NH_4_HCO_3_. The eluate from the column was utterly concentrated with a rotary evaporator.

The pellet was dissolved in distilled H_2_O and was then put onto a Sephadex LH-20 gravity column (40 × 1400 mm gel bed) eluted with distilled H_2_O (flow: 200 mL h^−1^; fractions: 10 mL) to generate many fractions. Based on the ^31^P NMR analysis of each fraction, fractions with the same or similar ^31^P NMR profile were combined. All fractions were concentrated with a rotary evaporator. The step can be repeated several times by changing the different sizes of the Sephadex LH-20 column.

The fraction containing the target compound was dissolved in distilled H_2_O and further purified using a 10 × 250 mm HILIC column (Atlantis^®^HILIC Silica, 5 µm) and a gradient elution, yielding pure **1**. The gradient used was: 20 min at 100% solvent B (0.1% TFA in ACN) then a linear gradient to 60% solvent A (0.1% TFA in distilled H_2_O) over 40 min. The flow rate was 3 mL min^−1^.

### Statistical analysis and data visualization

All statistical tests were computed in the Sigmaplot 12.5, and data visualization was performed using the package ggplot2 in R software v.3.1.3.

### Reporting summary

Further information on research design is available in the Nature Research Reporting Summary linked to this article.

## Supplementary information

Supplementary Information

Description of Additional Supplementary Files

Supplementary Data 1

Reporting Summary

## Data Availability

Genome sequence data of *S. monomycini* NRRL B-2409 has been deposited in the National Center for Biotechnology Information (NCBI) GenBank database with the BioProject accession JAFIQY000000000. The source data underlying Figs. 5b and 7b are provided as a Source Data file. Source data are provided with this paper.

## Source data

Source Data
