# Supplementary material for: Harnessing phosphonate antibiotics argolaphos biosynthesis enables a synthetic biology-based green synthesis of glyphosate
Source: Nat Commun. 2022 Apr 1;13:1736. doi: 10.1038/s41467-022-29188-6 (PMC8976061; doi:10.1038/s41467-022-29188-6)
Supplement: Supplementary file 1 — Supplementary Information [file 41467_2022_29188_MOESM1_ESM.pdf]

Harnessing phosphonate antibiotics argolaphos biosynthesis enables a synthetic biology-based green synthesis of glyphosate

Chu *et al*.

Supplementary Table 1. Plasmids or strains used in this study.

Name	Features	Source
**Plasmids**		
pET-15b	Amp^R, *E.coli* N-terminal 6 ×His-tag® expression vector	Novagen[1]
pAE4	*E.coli*/*S.lividans* shuttle vector<br>Apr^R, *ori*T, *ori*R6K, ΦC31*attP*, ΦC31*int*, *λattP* vector	
pRS416	Yeast centromere vector with a URA3 marker and an MCS derived from pBLUESCRIPT II.	New England Biolabs
pET15b-AlpG	pET-15b vector encoding for AlpG.	This study
pYES	Vector containing CEN6, ARS H4, URA3, oriR6K, accIV, oriT, ΦC31*attP* , ΦC31*int*, *λattP*, and *tL3*.	This study
pYES-*alpA*-*O*	pYES containing the *alp* gene cluster with the orignial promoter and RBS.	This study
pYES-*gapdh*p(EL)-*alpA*-*O*	pYES containing the *alp* gene cluster with the promoter gapdhp (EL) and the corresponding RBS.	This study
pYES-*ermE**p-*alpHI*	pYES containing the genes *alpHI* with the promoter ermE*p and the corresponding RBS.	This study
pYES-*ermE**p-*alpHIJ*	pYES containing the genes *alpHIJ* with the promoter ermE*p and the corresponding RBS.	This study
pYES-*ermE**p-*alpGHIJ*	pYES containing the genes *alpGHIJ* with the promoter ermE*p and the corresponding RBS.	This study
pYES-*ermE**p-*alpGHIJK*	pYES containing the genes *alpGHIJK* with the promoter ermE*p and the corresponding RBS.	This study
pYES-*ermE**p-*alpGHIJKL*	pYES containing the genes *alpGHIJKL* with the promoter ermE*p and the corresponding RBS.	This study
pYES-*ermE**p-RiboJ-*alpGHIJ*-*gapdh*p(EL)-SarJ-*KL*	pYES containing the genes *alpGHIJ* with the ermE*p-RiboJ-RBS cassette and the genes alpKL with the gapdhp(EL)-SarJ-RBS cassette	This study
**Strains**		
Escherichia coli DH5α/λpir	F-, *sup E44*, *recA1*, *endA1*, *hsdR17*, *thi-1*, *gyrA96*, *relA1*, *deoR*, *△(lacZYA-argF)-U169*, *Φ80dlacZ△M15*	[2]
E. coli Rosetta 2 (DE3) pLysS	F^-, *omp*T, *hsd*S_B($r_B^-m_B^-$), *gal*, *dcm* (DE30/ pLysSRARE(Cam^R)	Novagen
Escherichia coli BW25141	*lacI*^{q}, *rrnB*$_{T14}$, △*lacZ*$_{WJ16}$, △*phoBR580, hsdR514,* △*araBAD*$_{AH33}$, △*rhaBAD*$_{LD78}$, *galU95, endA*$_{BT333}$, *uidA* (△*MluI*)::*pir*$^+$ *recA1*, derived from E. coli K-12 strain BD792	[3]
E. coli WM6026	*lacI*^{q}, *rrnB3*, *△lacZ4787,hsdR514,△araBAD567,△rhaBAD568*, *rph-1*, *attλ*::pAE12 (△*ori*R6K-*cat*::Frt5), △*endA*::Frt, *uidA*(△MluI)::*pir*, *att*HK::pJK1006△(*ori*R6K-*cat*::Frt5; *trfA*::Frt)	[4]
Saccharomyces cerevisiae HZ848	*MATα*, *ade*2-1, △*ura*3, *his*3-11, 15, *trp*1-1, *leu*2-3, 112 and *can*1-100	[5]
Streptomyces lividans 66	Wild type (NRRL B-16148)	ARS
Streptomyces monomycin NRRL B-24309	Algolaphos A-producing strain	ARS

Supplementary Table 2. ^{1}H **(400 MHz),** ^{13}C **(100 MHz) and** ^{31}P **(161 MHz) spectroscopic data for compound 8 in** D_2O**.**

No.	δ_C, mult	δ_H (J in Hz)
1	37.5, CH_2	3.22, 3.05, m
	(J=100.0 Hz)	
1'	172.6, qC	-
	(J=12 Hz)	
2'	53.8, CH	4.23, t (7.0)
3’	28.1, CH_2	1.67, m
4’	24.2, CH_2	1.51, m
5'	40.4, CH_2	3.05, m
6'	156.8, qC	-
1"	169.4, qC	-
2"	58.3, CH	3.71, d (6.6)
3’’	30.0, CH	2.07, m
4’’	17.6, CH_3	0.86, m
5’’	17.8, CH_3	0.86, m
P	13.0, t (J=13.0 Hz)	-

Supplementary Table 3. Amino acid sequences of six MPnS-related proteins used in this study.

>G0051B_ GM000266 HepD *Streptomyces monomycini* NRRL B-24309<br>MSVPDPAFDRRAAALLRAAANDLKRDDTAAEADLGLAPGTFAGLTSGAQPVDLALLGRAAE<br>VWPLNERDLLPGHDDTSRRVRVMRAKESEASSRVLARGGDDYYEYRDTAMSRVASYRPEWI<br>RMLQPVDDNDADNPAVRWNRGHLLYQFTYFVGPVNYYYRWQDRSVCVPMETGDSVWGLPF<br>APHSFTARSTDEPAYILALTYGGDLVGDAQRELAILGNAAAHRAKLSTEAGPAALLRSFLEAR<br>AVTVEELARRSGLPAGHLAALVAGDRPPSDAERAALAEALGVSERDLLPPSTRTDGGVVVQR<br>SATAHRWNYPADAPAYRFTQLAGDASHPHTTALETEVLARSADDTAWLSTYQHSYLYVLGD<br>QPVRLRWEADGEQYDEELQPGDSAYVMPEVPLSLVRADPQAAPARVLLLRIAGAVLPEVRYA<br>LGAMPDGGIERYVNEDRLWYSKEGN
>WP_033205181.1 HppE *Streptomyces wedmorensis* NRRL 3426<br>MSNTKTASTGFAELLKDRREQVKMDHAALASLLGETPETVAAWENGEGGELTLTQLGRIAHV<br>LGTSIGALTPPAGNDLDDGVIIQMPDERPILKGVRDNVDYYVYNCLVRTKRAPSLVPLVVDVL<br>TDNPDDAKFNSGHAGNEFLFVLEGEIHMKWGDKENPKEALLPTGASMFVEEHVPHAFTAAKG<br>TGSAKLIAVNF
>WP_003988638.1 Class I HepD *Streptomyces viridochromogenes* DSM 40736<br>MRIDPFKLAHWMNARKYTAAQTADLAGLPLDDLRRLLGDEANEPDPAAATALAEALSVEPS<br>QLAADAHRNLTVVHKSAEEMHASRRPIQRDGIHFYNYYTLAAPEGRVAPVVLDILCPSDRLPA<br>LNNGHLEPAITVNLGPGDINGRWGEEITPQTWRVLHANHGGDRWITGDSYVEPSYCPHSYSLA<br>GDAPARIVSYTAQSNISPLMTEANNWSTGAFEEALKALSGKVSAGSVLDLFLARRAHTRTSAA<br>EAAGVPPADLEAALRSPASETGLTVLRTLGRALGFDYRVLLPADDQHDGVGKTWTTIEDSRRS<br>RRTFGTYEAASMASAAHLPDLVGSFLRVDADGRGADLIDHAENHYVVTEGRLTLEWDGPDG<br>PASVELEPDGSAWTGPFVRHRWHGTGTVLKFGSGAHLGYQDWLELTNTFEPAATLRRGRRDL<br>AGWGYDN
>WP_032782080.1 Class II HepD *Streptomyces albus* NRRL B-16041<br>MSTPDALGSDAARAARAAALLRAAANDLKRNDRAAEADLGLPPGSFGDYVSGRLPITWDLIS<br>RAAQAWPLNERDLLPIHNDTPQGLRMMRVKESEASSRIIERGGGPYYEYRDTAMSRQASYRPE<br>WISMLRVVEDDDPDNPLVEWNKGHLLYQFTYFVGPVNYYFRSGGRSHCVPMNTGDSVWGLP<br>FAPHSFTARSADEPAYILALTYGGELTGDAQRELATFGRAVTSSLALTPGDHGAMLRSVMAAR<br>LTTVTELADRSGLKTDRVAALCRTPARAEWPELSALAEALGVSVRELLVPHTTTEADVRIQPG<br>RTASRWSYPGPDAPAYRFTQLAGDPLHPHTTSLAVDVLTARPDAPLPPTYQHQYLYVLGEQP<br>VSVRWRYNGEQYDGRLEPGDSAYVIPGIEFSLSAEKPTELLMLRIGGSATPDVRFALGAMPDG<br>AIGRYIAEDRLWY
>WP_012214540.1 MPn *Candidatus Nitrosopumilus maritimus* SCM1<br>MEKKIDFKPDSYLIRSGNNFLGILNDIKRRPEDAANELGVSIEEINSIISGKQKISPSLIEKAVNIW<br>PVNERDFYIVSDDCSSGILIMTSQDSIKSSRIMERAGKPYYEYRDTAMSKTAPFRPEWILELCKV<br>ENNDPENPKAQWNNGHFMHQFTYFIGEVNFYYKDPEGKKHVAIMNTGDSMYITPFTPHTFTT<br>RDGASQNGLILALTYGSKLTGDIQQELSSLSLDCGSQYALDFTNHENASLSLLEYYFELSNLTK<br>EKFAKRTNFSMETLADFFTKKKLPTFDELKIIAKALNVNSRDLMPNDLTESKVIVKTHDQCDH<br>WKYPESGNYEFYELASTTALPHSKAFEIDVSSSEDLNLDLKVGLHQYVYNIGDSALTINWNYE<br>NKTYQKSLNPGDSAYIKPFVPHNFRGNGKILILRIGGKISGDSQRELSFVGRENTQRAISETMQ<br>WFDPKGSNS
>WP_029455269.1 MPn *Candidatus Pelagibacter ubique* HTCC7217<br>MSISNIKFRKILNDLKRRPEDAARDLKISNKKLLQILNNKTKTDFDIIEKATKIWPVNYGDFFSFE<br>DDTKNDFKIMRASISDKSSRIMSRGKKPYYLYKDTVMSKLSPFKPELITELQIVSDNYSNNIDV<br>KFNNGHFLHQFTYFIGPVNFYYMLNGKKKVAVMNTGDSMYISPYIPHSFATRKNNQGVLGKII<br>AITYSDKLDNETLNESSSIGFNLIKKLKVNLKDEYNSFWSNLEKQINNSFISFNMLNELLKYDLG<br>SLKKNKKIPKINTIKKIAKYLNLNLRDLLPPNNLTDVKIQKYKDNRSWFYPSNTKRDYKIIELTN<br>VSELPYSRGFEIKILKNNKKNCFLEVPTHQYIYNIGKKDIKIKIDGINEKLNKNDSMYIKPNKKH<br>KFISEGKVLVLRLGGRLSGDSLYQLSKMSDKNLKRTLNDNKPWFNK

Supplementary Table 4. Amino acid sequence similarity and identity of AlpG, SwHppE, SvHepD, SaHepD, NmMPnS and CpuMPnS.

	AlpG	SwHppE	SvHepD	SaHepD	NmMPnS
AlpG	100/100 [a]	17.67/13.13	14.44/7.22	18.22/14.44	13.4/7.25
SwHppE	17.67/13.13	100/100	12.12/7.07	8.58/4.04	8.08/6.06
SvHepD	14.44/7.22	12.12/7.07	100/100	12.86/7.9	10.83/5.41
SaHepD	18.22/14.44	8.58/4.04	12.86/7.9	100/100	18.22/11.77
NmMPnS	13.4/7.25	8.08/6.06	10.83/5.41	18.22/11.77	100/100
CPuMPnS	11.98/5.06	11.61/8.08	15.43/8.28	12.21/4.83	12.67/6.22

[a] Similarity/Identity.

Supplementary Table 5. The DNA sequences of promoters used in this study.

Promoter	Sequence (5’->3’)	Resource [a]
P1	TGTTCACATTCGAACCGTCTCTGCTTTGACAACATGCTGTGCGGTGTT<br>GTAAAGTCGTGGCC	*kasO*p*[6]
P2	GCTGCTCCTTCGGTCGGACGTGCGTCTACGGGCACCTTACCGCAGCCG<br>TCGGCTGTGCGACACGGACGGATCGGGCGAACTGGCCGATGCTGGGA<br>GAAGCGCGCTGCTGTACGGCGCGCACCGGGTGCGGAGCCCCTCGGCG<br>AGCGGTGTGAAACTTCTGTGAATGGCCTGTTCGGTTGCTTTTTTTATA<br>CGGCTGCCAGATAAGGCTTGCAGCATCTGGGCGGCTACCGCTATGAT<br>CGGGGCGTTCCTGCAATTCTTAGTGCGAG	*gapdh*p (EL)[5]
P3	CCCGCCGCGGGCGCTGGAGGCTCGGGCGGGCCCCGGGCCGGAGGCG<br>GCCGCGACCACGACGCCCGCGGGACGTGACGAGCGGCACGACTCGA<br>CGACTCCGGGCTCCTTTGACGCTGTCCGTCGCGCCGGGTAGCGTAGG<br>ACACCGTGCCCGCGCCGTCGGGCCCTCGCGCGTGCACTCGGTCGACC<br>GCTCCCTGCCGGAGTGGGTGCGGGTGCACGGGGTGGCTCCCCACCTC<br>CTCTCGGATCGGTCCTCGCGGACTGCCGCCGTGCGGAGGACCGGGGC<br>GACACGCCCGGGCGCGGGGGTCGGTGCGGGACTCCAGACCTCCGGG<br>GTAGTCGTGCGACGGGCGACGATCCGGGCCGAGCCGGCCGTCCTGGG<br>TGACGGGTGCCGGTCAGACCAG AGAA	*rpsL*p (CF)[5]
P4	CACGGGCCGGTGAACCCCGCATGAATGTTGGTTCATGTGGGGTTTGC<br>GCCAGCTGTGTCGCAGTTGCCCCCCGCCACTGTTCCCGGGGCATGAAT<br>CCCGCTAGAATGAACACGGAAGAGCAGGTGGACCCCGGGTCGTCACC<br>GCACTTCGGGTGTGGGATTTATGCCGATTGGTTTTGATCCCACATCCG<br>GACGGGCCGGAGTGCACCGCACACC	*gapdh*p<br>(KR)[5]
P5	TCCGTTCCCGGCGGGGAGCGGGACACCCGGGGAGTTCGCCCGGAAAT<br>GTCCGAAGCTAGGCGAAGAAAGTGTGACGAGCACGAAGTCCCTGCTC<br>CCTGCCGGTGCGAATGTGCCACGCGATAGGGTGTGGTGACAGGCACA<br>C	*gapdh*p<br>(TP)[5]
P6	AGGGGCGCGCGGCCCCGGCCGCCGGCCGTCCCGTCCGCGGGCCCGTG<br>GCCGGGGCGGTCCCGGCGTGTCGCGGCGGACAGCATTTGTTTTGACC<br>CAGCTCCGTGAGGTAGGTACGCTCAAGCCTTGTGCCTGGGGTGTGCC<br>TGGGCTCGGGTGCGTGTCCTCAACCGCATCGCGAGTCCGTCAGTAGC<br>CACCGCAATCTGCGCCCTTCCTGCCTTCGGGGCGGGAGTCCGCAGTAT<br>TCGACACACCCGACCGCGTGGGTCGGCGATGTTCCAGGTTAGTTTCA<br>CGAACGGC	*rpsL*p<br>(SG)[5]
P7	CTCGGCGGCGAGCGGGGGACGTTGGTCCCGCACCCGCCGGGACCCGG<br>GTCCCGGCGGGCGAAACGGAGGTCCGTGCTCCTACTGGCGAGTACGG<br>ATCTCCGGCCGTATCCGGTGGCGTGCGGTGGCCCTCTCGATGTCACCC<br>CCGGGGCCTCGGAGAGGTAGGGTCGGAAGCGGTCGGGGACATCCCT<br>ATACAACTCGCCGGCGTCGAAAACCGGCGTA	*gapdh*p<br>(SG)[5]
P8	CCTGCCCTCCGTCAGGGTGGGCTGCGCACGTTCGGGCCACGCGCATT<br>AGGGTATGAGTCACATCGACCGATGAACCCCACGTCCGCGTCGTGCA<br>CACACGGATTCGGAGTATGCGTGGGTCAAGTTGCACACCAGAAT	*gapdh*p<br>(RE)[7]
*ermE*p*	GAGCGAGTGTCCGTTCGAGTGGCGGCTTGCGCCCGATGCTAGTCGCG<br>GTTGATCGGCGATCGCAGGTGCACGCGGTCGA	*ermE*p*

[a] RE, *Rhodococcus erythropolis*; SG, *Streptomyces griseus*; KR, *Kocuria rhizophila*; TP, *Tsukamurella paurometabola*; CF, *Cellulomonas flavigena*; EL, *Eggerthella lenta*.

Supplementary Table 6. DNA sequences of ribosome binding sites (RBS) used in this study.

RBS	Sequence (5'->3')	Resource
R1	AGATTTTTTCGGGGGTGACC	Terminase (φC31)[7, 8]
R2	TTCTCGCGGGTGCTGCCTGG	dCMPdeaminase (φC31)[7, 8]
R3	CACAAGGGGTTGTGACCGGG	Integrase (φC31)[7, 8]
R4	TCTAAGTAAGGAGTGTCCAT	Major capsid protein (φC31)[7, 8]
R5	CGACGACCTAGGGGTTTTGC	Major tail protein (φC31)[7, 8]
R6	TGAGTGAAGGGAGTTGCCAC	DNA polymerase (φC31)[7, 8]
R7	TCATGACGTAAGGGGGCGCT	Tail tape measure protein (φC31)[7, 8]
R8	AACTACGAAGGGGAGTCAGT	Helicase (φC31)[7, 8]

Supplementary Table 7. DNA sequences of ribozyme-based insulators used in this study.

Name	Sequence (5’->3’) [9, 10]
SarJ	AGACTGTCGCCGGATGTGTATCCGACCTGACGATGGCCCAAAAGGGCCGAAACAGTCCTCTACAAATAATTTTGTTTAA
RiboJ	AGCTGTCACCGGATGTGCTTTCCGGTCTGATGAGTCCGTGAGGACGAAACAGCCTCTACAAATAATTTTGTTTAA

Supplementary Table 8. The composition of culture media used in this study.

Names	Compositions	Amount (g L^{-1})
MYG		
	Malt extract	10
	Yeast extract	4
	D-glucose	4
YPAD		
	Yeast extract	10
	Peptone	20
	Dextrose	20
	Adenine hemisulphate	0.16
Luria Broth (LB)		
	Tryptone	10
	NaCl	10
	Yeast extract	5
	pH	7
ATCC172		
	D-glucose	10
	Soluble starch	20
	Yeast extract	5
	N-Z amine type A	5
	$CaCO_3$	1
ISP2		
	Yeast extract	4
	Malt extract	10
	D-glucose	4
ISP4		
	Soluble starch	10
	$CaCO_3$	2
	$(NH_4)_2SO_4$	2
	K_2HPO_4	1
	$MgSO_4·7H_2O$	1
	NaCl	1
	$FeSO_4·7H_2O$	0.001
	$MnCl_2·7H_2O$	0.001
	$ZnSO_4·7H_2O$	0.001
SOC		
	Tryptone	20
	Yeast extract	5
	NaCl	0.5
	KCl	0.186 (2.5 mM)
	$MgCl_2$	0.96 (10 mM)
	$MgSO_4$	1.2 (10 mM)
	D-glucose	3.6 (20 mM)

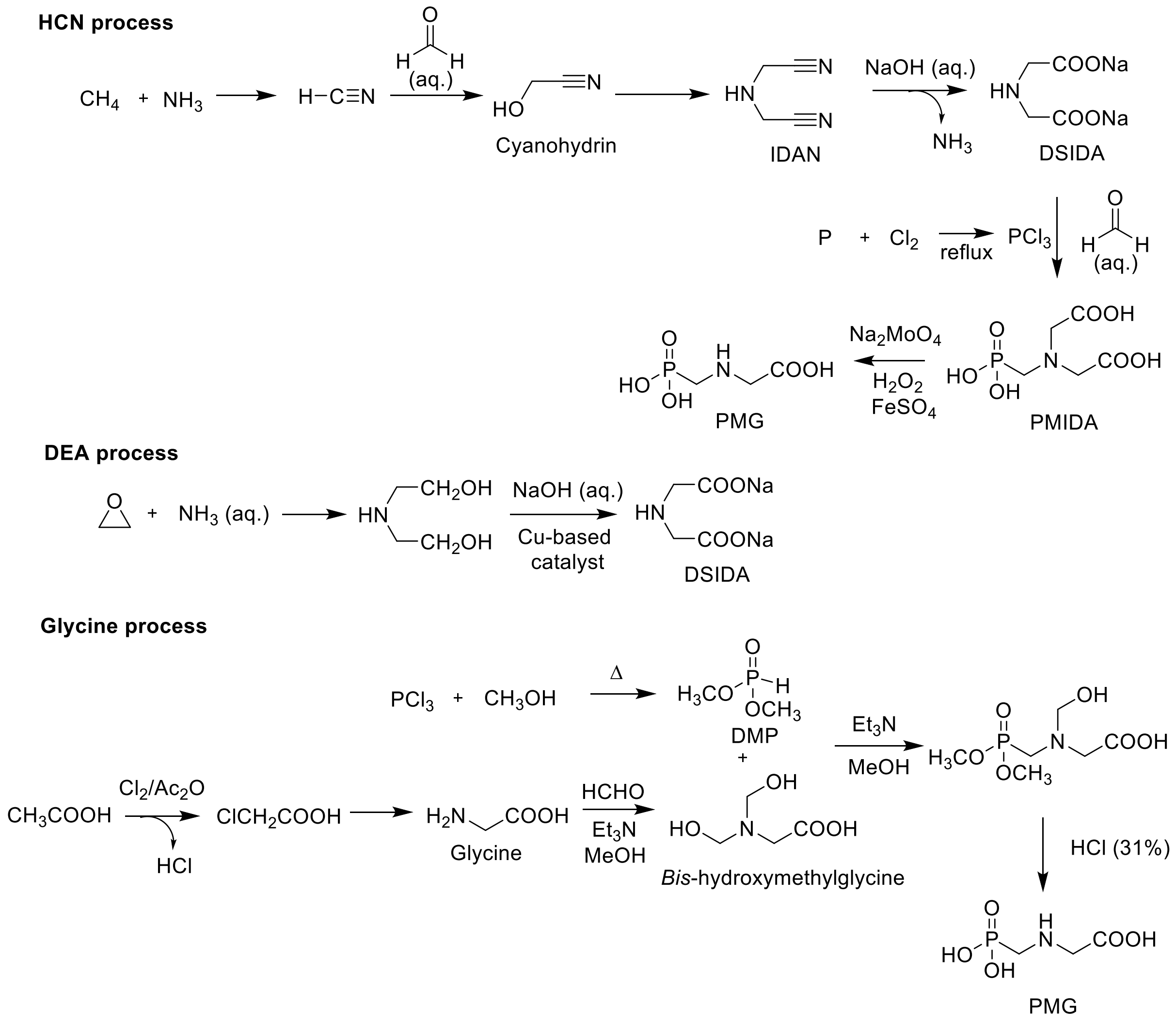

Supplementary Fig. 1. A schematic diagram of the three commercialized processes of glyphosate.

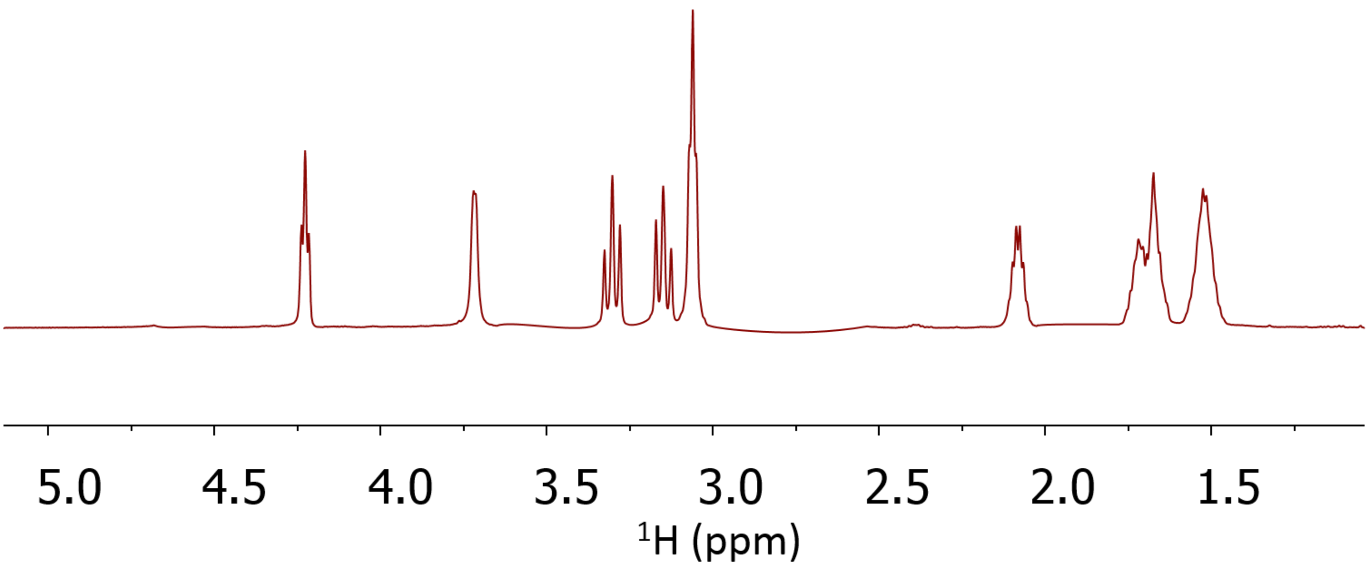

Supplementary Fig. 2. ^{1}H spectrum of compound 8 in D_2O.

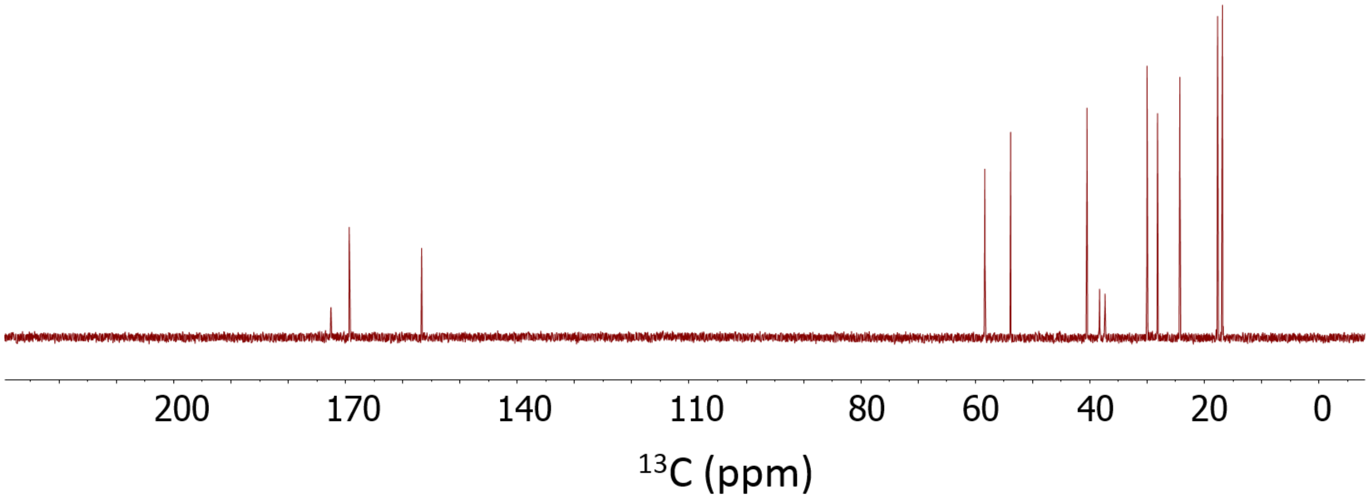

Supplementary Fig. 3. ^{13}C NMR spectrum of compound 8 in D_2O.

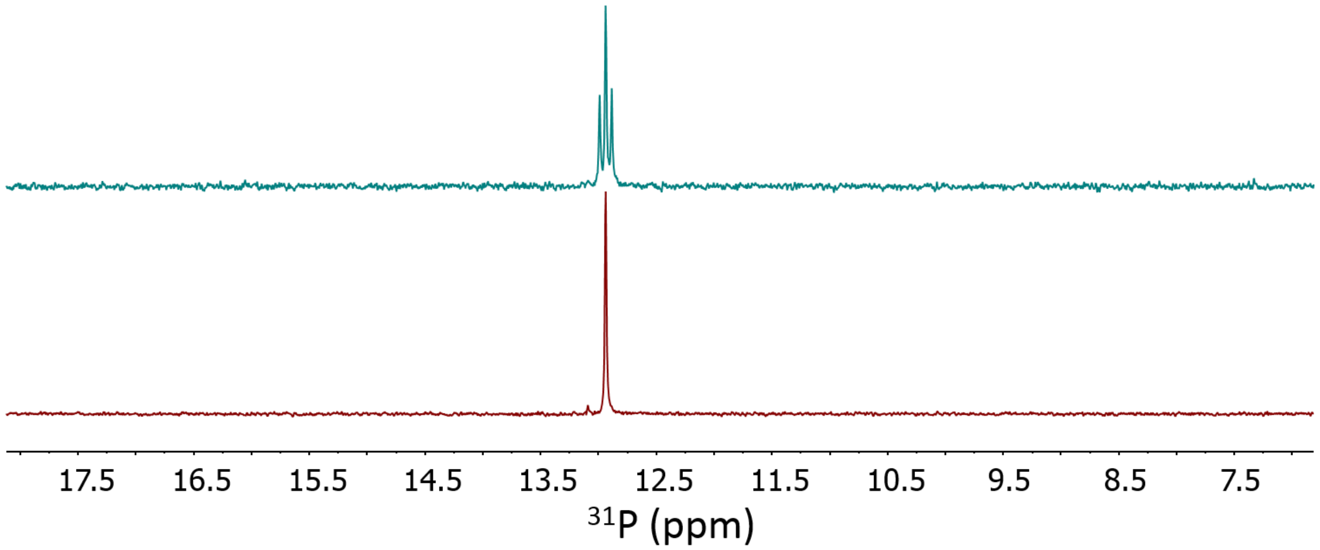

Supplementary Fig. 4. ^{1}H-decoupled- and ^{1}H-coupled-^{31}P NMR spectra (bottom and top) of compound 8 in D_2O.

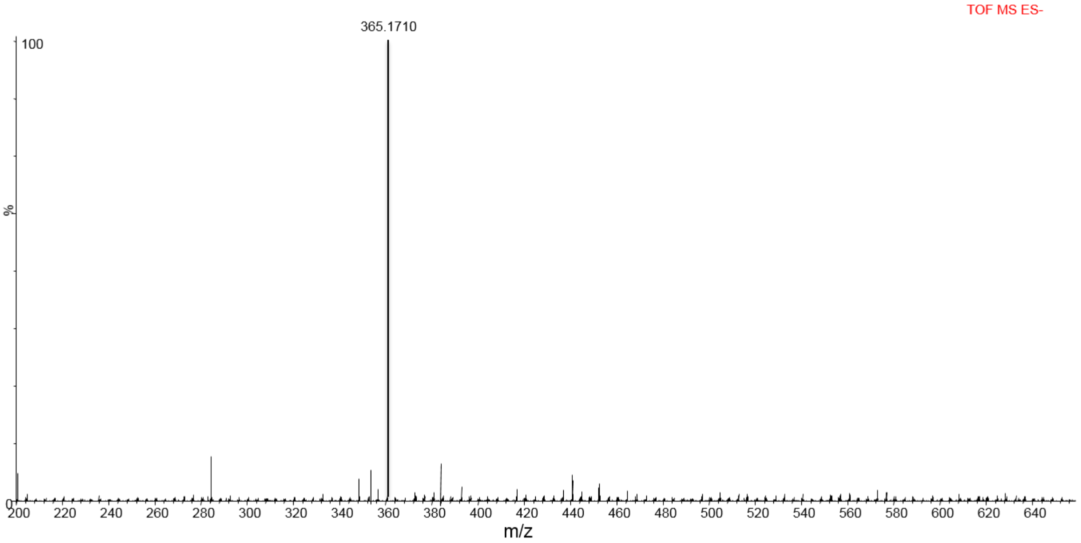

Supplementary Fig. 5. High-resolution mass spectrometry of compound 8.

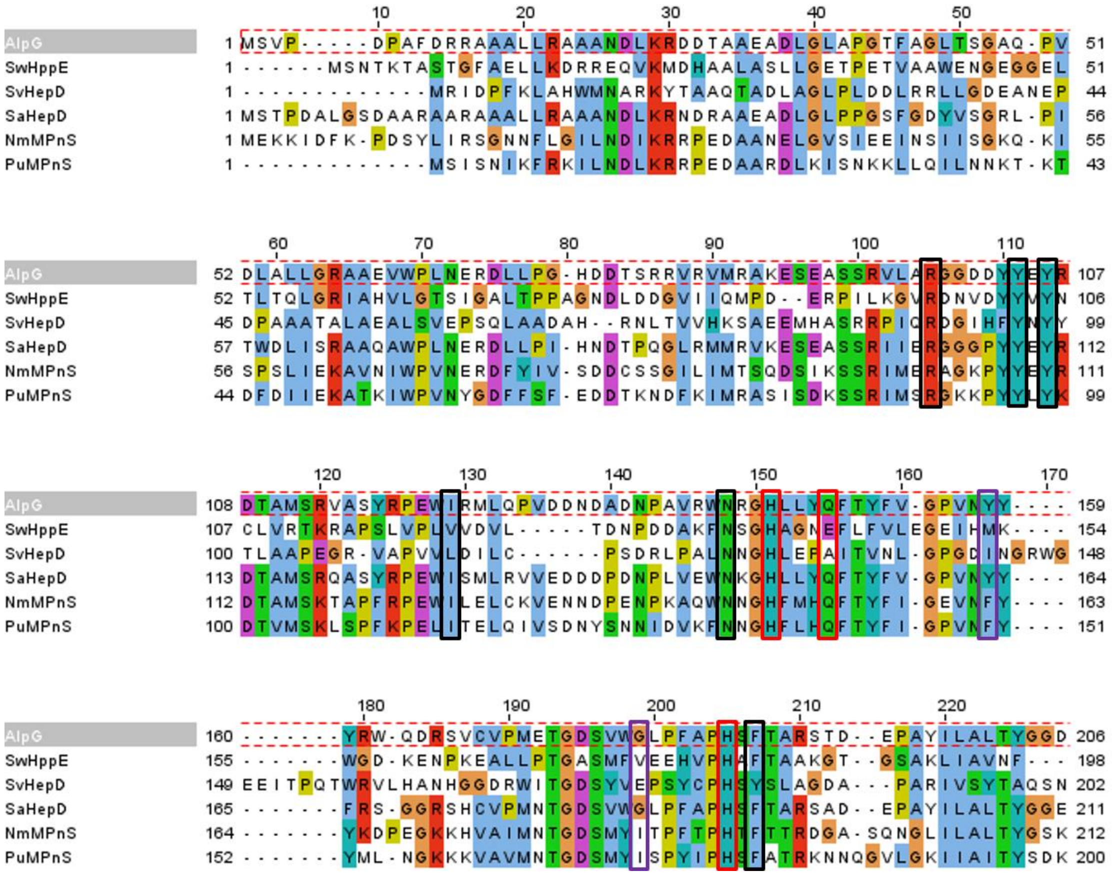

Supplementary Fig. 6. Sequence alignment of the N-terminal region that contains a cupin fold and the active site residues of MPnS-related proteins. Iron-coordinating residues are highlighted in red frames. A multiple sequence alignment of 6 discrete MPnS-related proteins was generated using the Clustal Omega[11] web tool (http://www.ebi.ac.uk/Tools/msa/clustalo/) with the default parameters. Substrate-binding residues are highlighted in black frames. For enzymes with structurally similar active sites, the Gly-Tyr or Ile-Phe pairs that determine reaction pathways are highlighted in purple frames. AlgG (WP_030019525.1) from *Streptomyces monomycini* NRRL B-24309. SwHppE (WP_033205181.1) from *Streptomyces wedmorensis* NRRL 3426. SvHepD (WP_003988638.1) from *Streptomyces viridochromogenes* DSM 40736. SaHepD (WP_032782080.1) from *Streptomyces albus* NRRL B-16041. MPnS (WP_012214540.1) from *Candidatus Nitrosopumilus maritimus* Nitrosopumilus maritimus SCM1. MPnS (WP_029455269.1) from Candidatus Pelagibacter *ubique* HTCC7217.

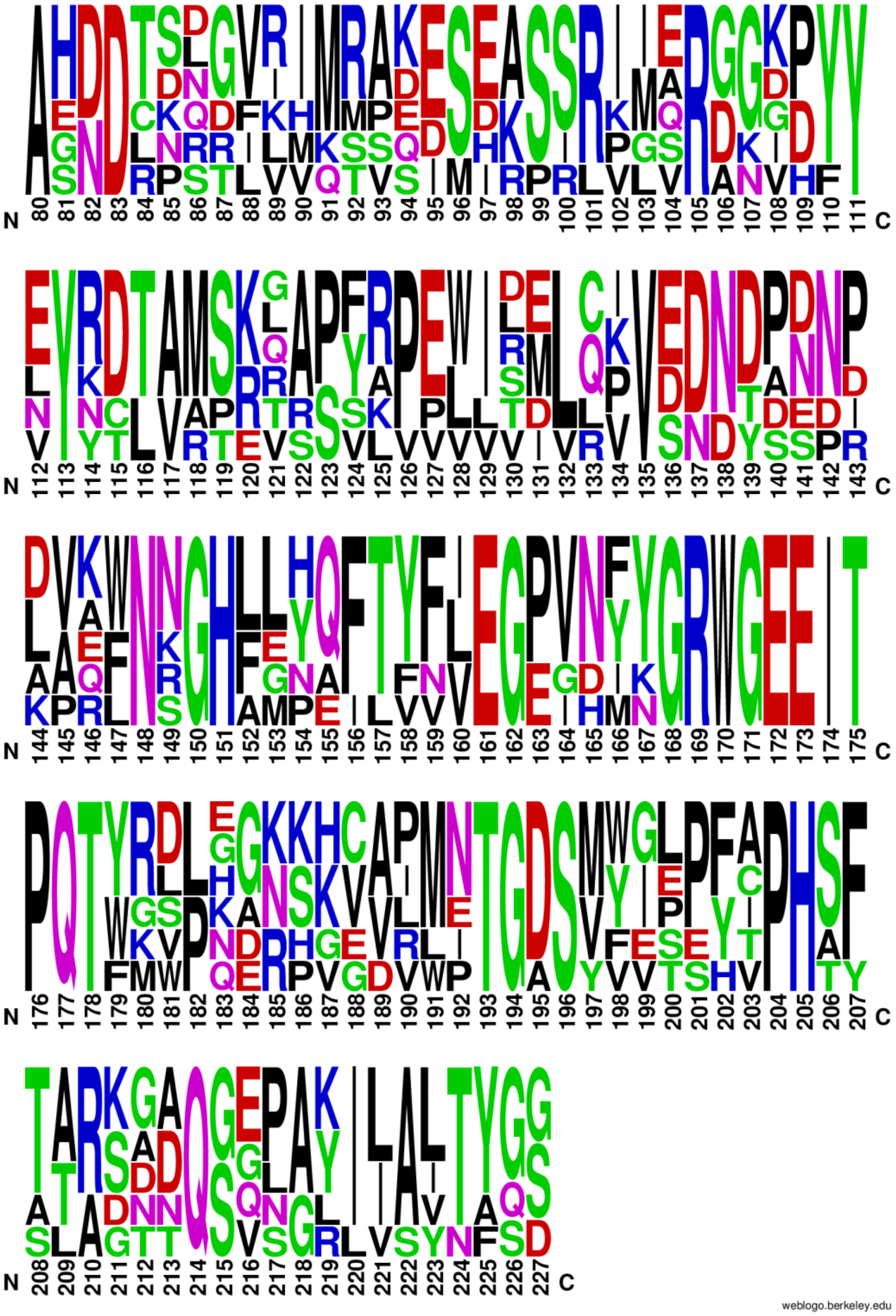

Supplementary Fig. 7. Sequence logos for MPnS-related protein families with six members show wide variance in sequence composition and degree of conservation. Six members are: AlgG (WP_030019525.1);SwHppE (WP_033205181.1);SvHepD (WP_003988638.1) ;SaHepD (WP_032782080.1); MPnS (WP_012214540.1); MPnS (WP_029455269.1).

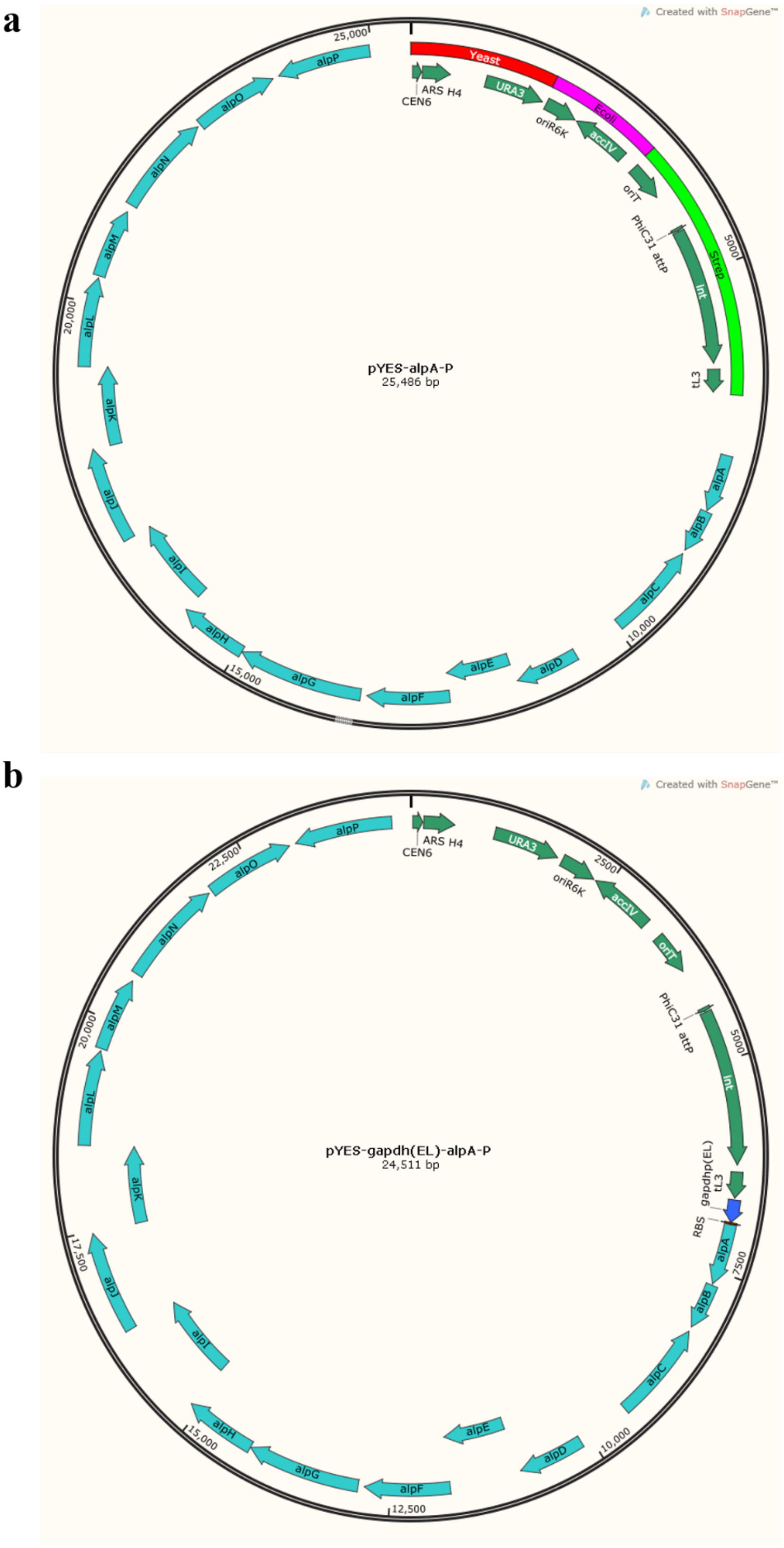

Supplementary Fig. 8. Vector maps of the construct carrying the alp gene cluster. (a) the alp gene cluster with the native promoter and RBS. (b) the alp gene cluster with the *gapdh*p (EL) and corresponding RBS.

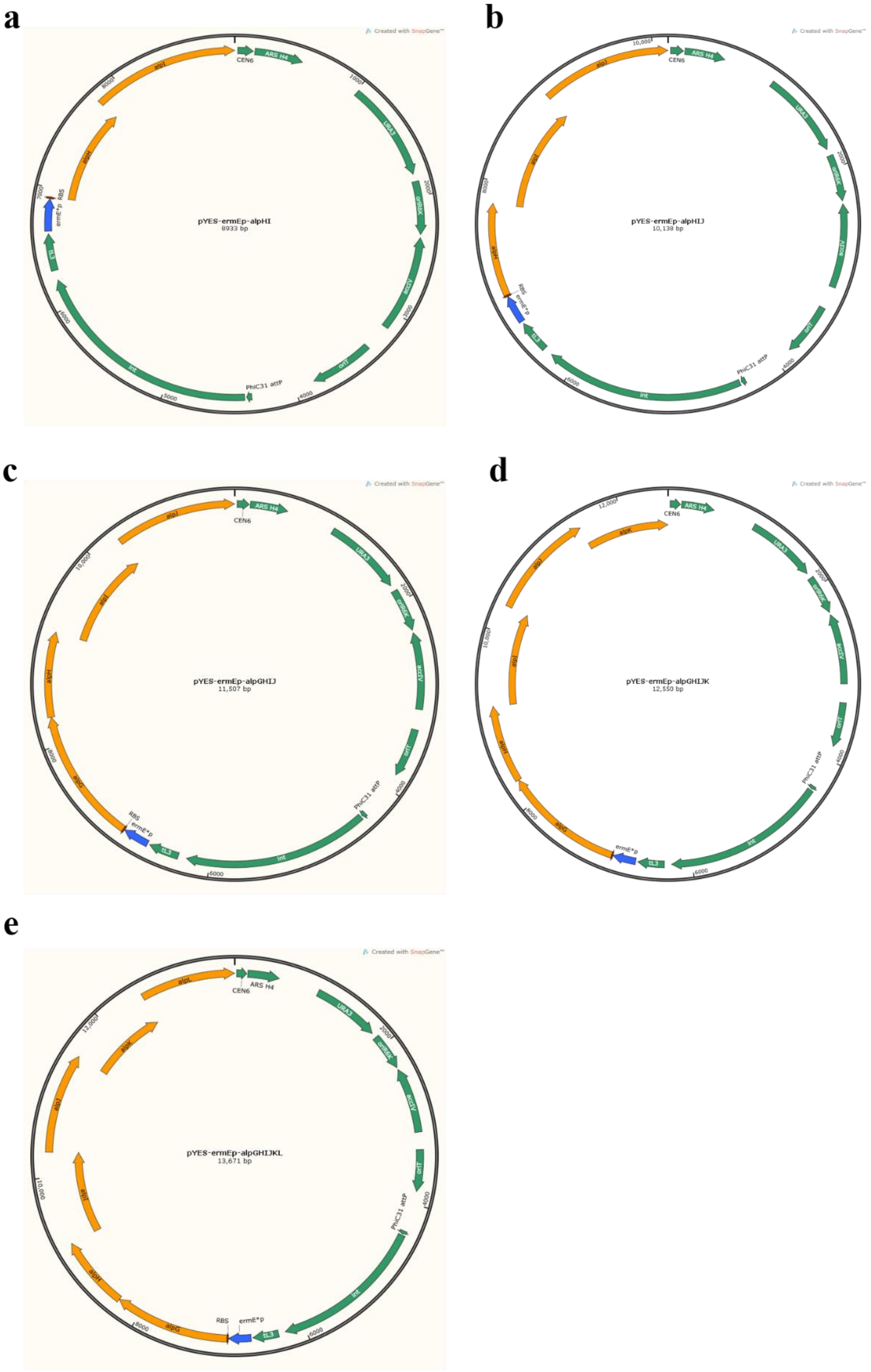

Supplementary Fig. 9. Vector maps of the ermE*p construct carrying the combination of different genes in the biosynthesis of AMP. (a) pYES-ermEp-alpHI containing the genes *alpHI* with the strong promoter ermE*p. (b) pYES-ermEp-alpHI containing the genes *alpHIJ* with the strong promoter ermE*p. (c) pYES-ermEp-alpHI containing the genes *alpGHIJ* with the strong promoter ermE*p. (d) pYES-ermEp-alpHI containing the genes *alpGHIJK* with the strong promoter ermE*p. (e) pYES-ermEp-alpHI containing the genes *alpGHIJKL* with the strong promoter ermE*p.

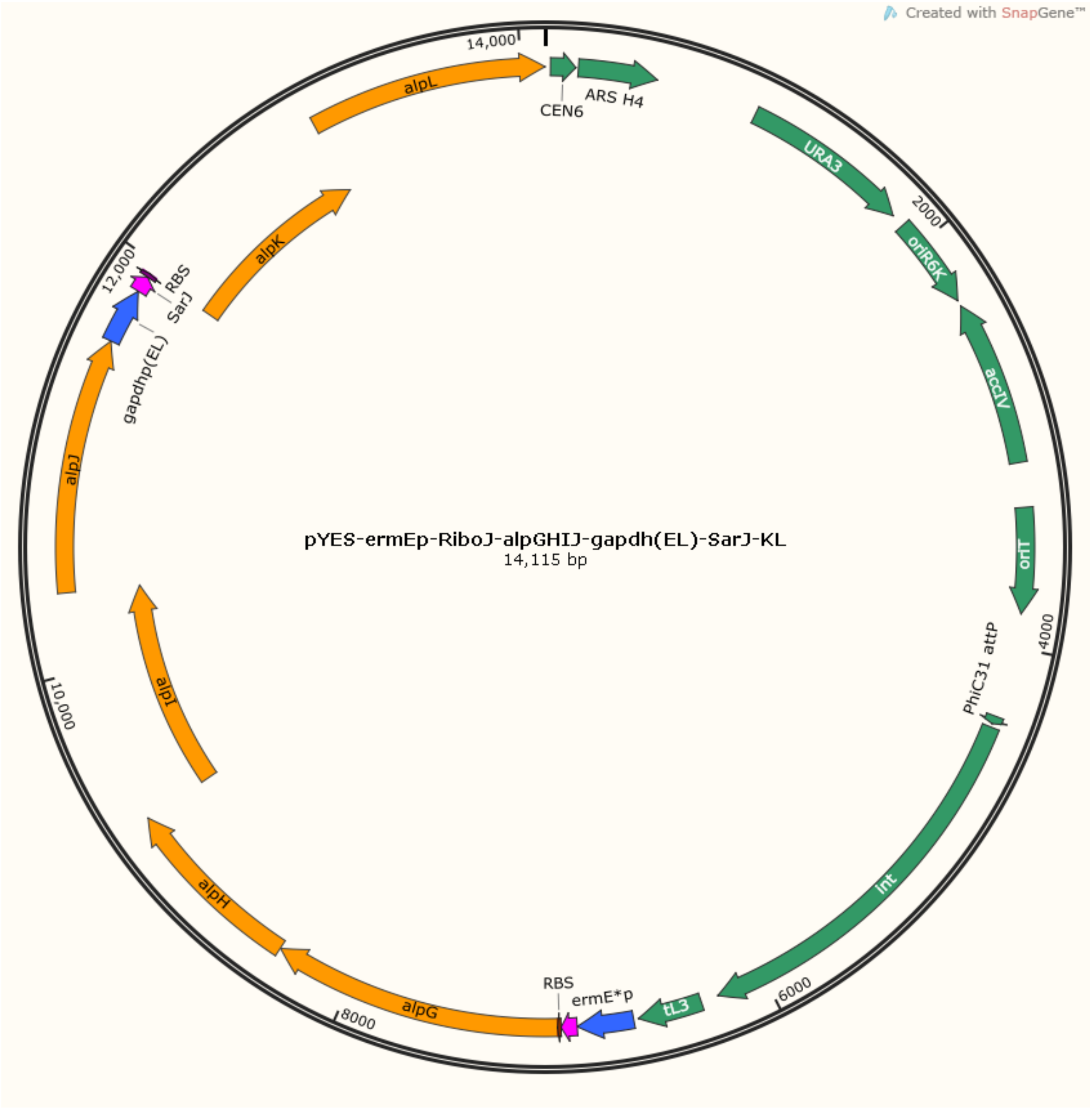

Supplementary Fig. 10. Vector map of the promoter-insulator-RBS construct carrying the genes alpGHIJKL in the biosynthesis of AMP.

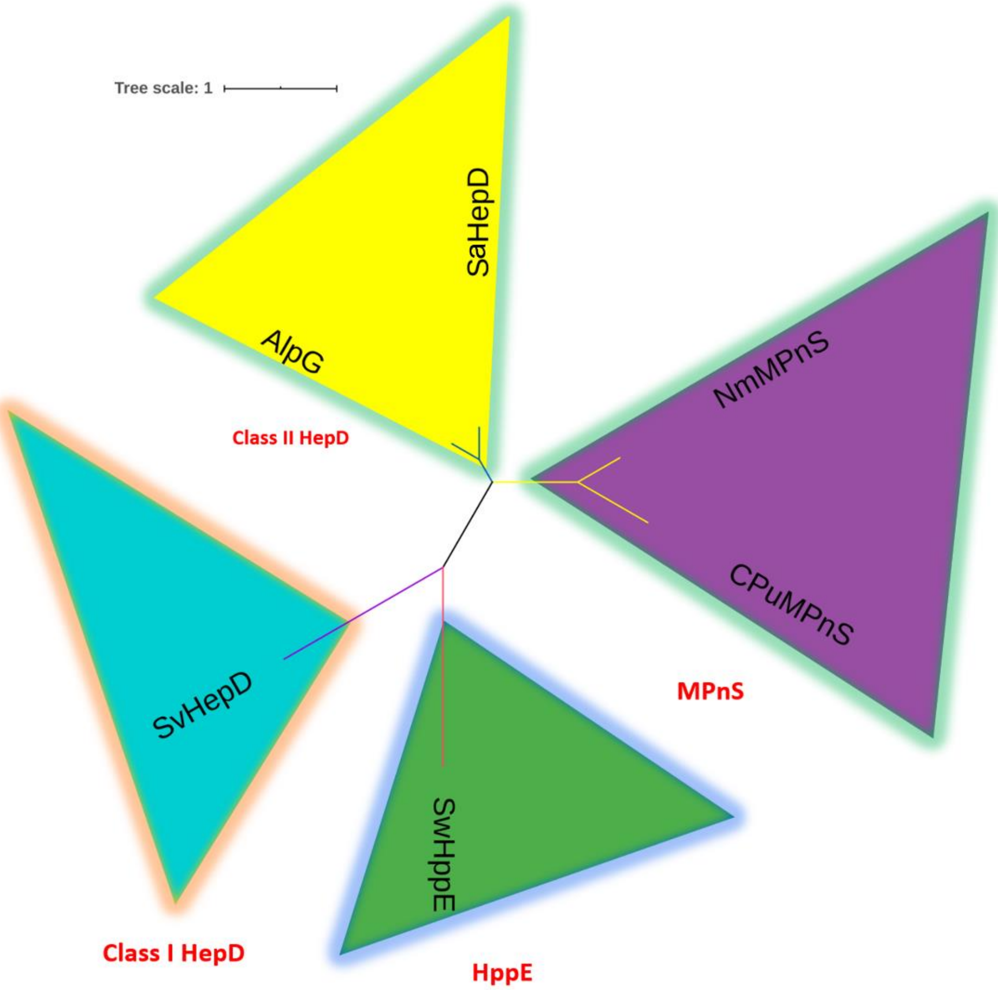

Supplementary Fig. 11. Phylogenetic tree analysis of AlpG and other MPnS-related proteins.

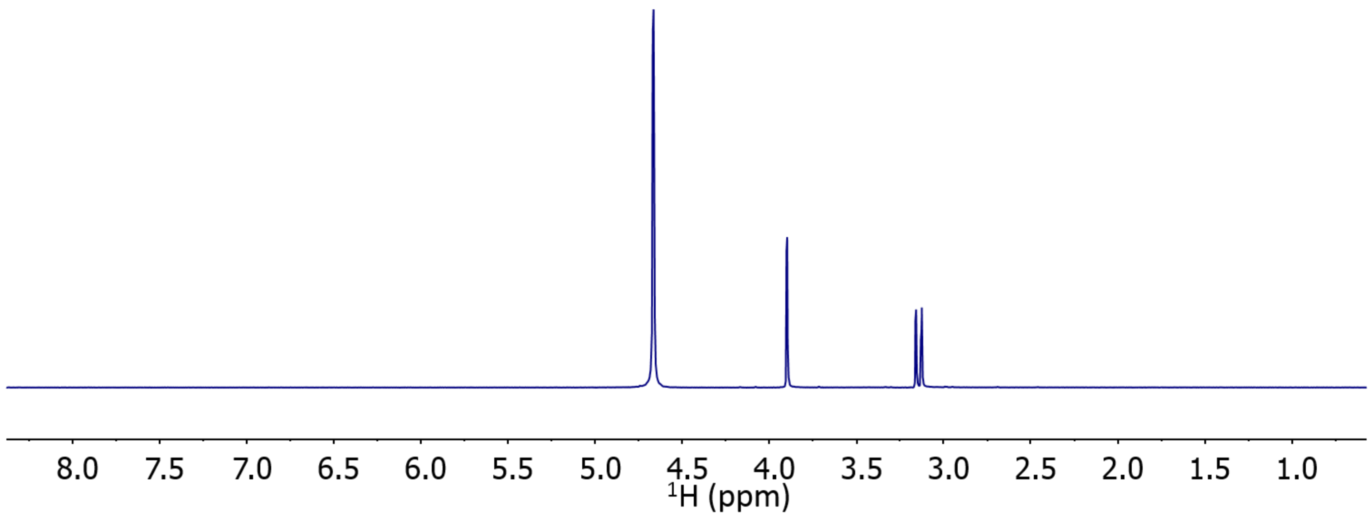

Supplementary Fig. 12. ^{1}H spectrum of glyphosate in D_2O.

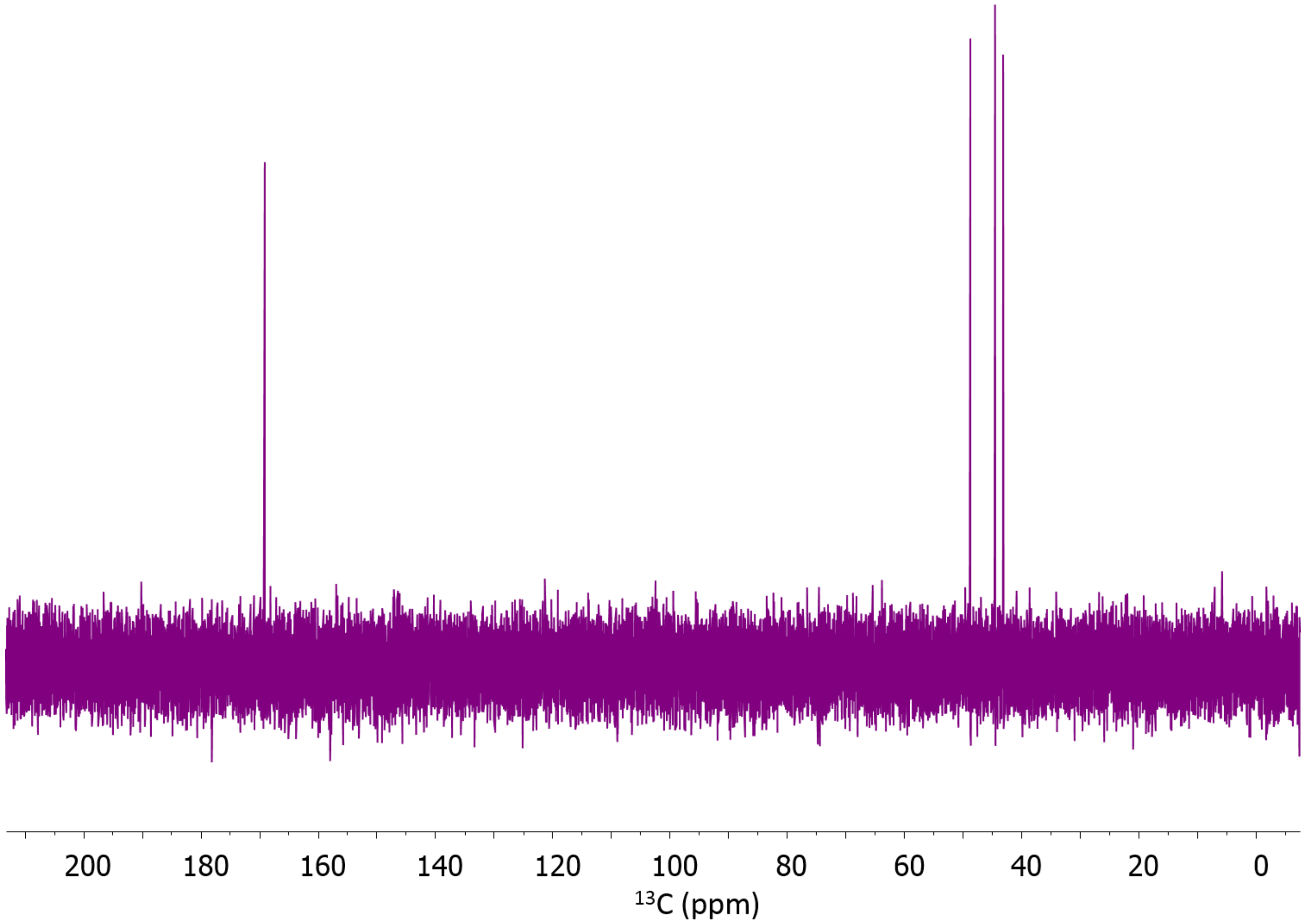

Supplementary Fig. 13. ^{13}C spectrum of glyphosate in D_2O.

Supplementary references

1. Woodyer RD*, et al.* Heterologous production of fosfomycin and identification of the minimal biosynthetic gene cluster. *Chem Biol* **13**, 1171-1182 (2006).

2. Grant SG, Jessee J, Bloom FR, Hanahan D. Differential plasmid rescue from transgenic mouse DNAs into Escherichia coli methylation-restriction mutants. *Proc Natl Acad Sci U S A* **87**, 4645-4649 (1990).

3. Datsenko KA, Wanner BL. One-step inactivation of chromosomal genes in Escherichia coli K-12 using PCR products. *Proc Natl Acad Sci U S A* **97**, 6640-6645 (2000).

4. Blodgett JA*, et al.* Unusual transformations in the biosynthesis of the antibiotic phosphinothricin tripeptide. *Nat Chem Biol* **3**, 480-485 (2007).

5. Shao Z, Rao G, Li C, Abil Z, Luo Y, Zhao H. Refactoring the silent spectinabilin gene cluster using a plug-and-play scaffold. *ACS Synth Biol* **2**, 662-669 (2013).

6. Wang W, Li X, Wang J, Xiang S, Feng X, Yang K. An engineered strong promoter for streptomycetes. *Appl Environ Microbiol* **79**, 4484-4492 (2013).

7. Bai C*, et al.* Exploiting a precise design of universal synthetic modular regulatory elements to unlock the microbial natural products in Streptomyces. *Proc Natl Acad Sci U S A* **112**, 12181-12186 (2015).

8. Smith MC, Burns RN, Wilson SE, Gregory MA. The complete genome sequence of the Streptomyces temperate phage φC31: evolutionary relationships to other viruses. *Nucleic Acids Res* **27**, 2145-2155 (1999).

9. Khvorova A, Lescoute A, Westhof E, Jayasena SD. Sequence elements outside the hammerhead ribozyme catalytic core enable intracellular activity. *Nat Struct Mol Biol* **10**, 708-712 (2003).

10. Nelson JA, Shepotinovskaya I, Uhlenbeck OC. Hammerheads derived from sTRSV show enhanced cleavage and ligation rate constants. *Biochemistry* **44**, 14577-14585 (2005).

11. Sievers F*, et al.* Fast, scalable generation of high-quality protein multiple sequence alignments using Clustal Omega. *Mol Syst Biol* **7**, 539 (2011).
