# Supplementary material for: Harnessing phosphonate antibiotics argolaphos biosynthesis enables a synthetic biology-based green synthesis of glyphosate
Source: Nat Commun. 2022 Apr 1;13:1736. doi: 10.1038/s41467-022-29188-6 (PMC8976061; doi:10.1038/s41467-022-29188-6)
Supplement: Supplementary file 2 — Description of Additional Supplementary Files [file 41467_2022_29188_MOESM2_ESM.pdf]

Description of Additional Supplementary Files

File Name: Supplementary Data 1
Description: The primers used in this study.
