# Supplementary material for: Harnessing phosphonate antibiotics argolaphos biosynthesis enables a synthetic biology-based green synthesis of glyphosate
Source: Nat Commun. 2022 Apr 1;13:1736. doi: 10.1038/s41467-022-29188-6 (PMC8976061; doi:10.1038/s41467-022-29188-6)
Supplement: Supplementary file 4 — Reporting Summary [file 41467_2022_29188_MOESM4_ESM.pdf]

nature research

Corresponding author(s): Jiangtao Gao

Last updated by author(s): Feb 20, 2022

Reporting Summary

Nature Research wishes to improve the reproducibility of the work that we publish. This form provides structure for consistency and transparency in reporting. For further information on Nature Research policies, see our Editorial Policies and the Editorial Policy Checklist.

Statistics

For all statistical analyses, confirm that the following items are present in the figure legend, table legend, main text, or Methods section.

n/a	Confirmed	
☐	☒	The exact sample size (*n*) for each experimental group/condition, given as a discrete number and unit of measurement
☐	☒	A statement on whether measurements were taken from distinct samples or whether the same sample was measured repeatedly
☒	☐	The statistical test(s) used AND whether they are one- or two-sided<br>*Only common tests should be described solely by name; describe more complex techniques in the Methods section.*
☒	☐	A description of all covariates tested
☐	☒	A description of any assumptions or corrections, such as tests of normality and adjustment for multiple comparisons
☐	☒	A full description of the statistical parameters including central tendency (e.g. means) or other basic estimates (e.g. regression coefficient) AND variation (e.g. standard deviation) or associated estimates of uncertainty (e.g. confidence intervals)
☒	☐	For null hypothesis testing, the test statistic (e.g. F, t, r) with confidence intervals, effect sizes, degrees of freedom and P value noted<br>*Give P values as exact values whenever suitable.*
☒	☐	For Bayesian analysis, information on the choice of priors and Markov chain Monte Carlo settings
☒	☐	For hierarchical and complex designs, identification of the appropriate level for tests and full reporting of outcomes
☒	☐	Estimates of effect sizes (e.g. Cohen's *d*, Pearson's *r*), indicating how they were calculated

Our web collection on statistics for biologists contains articles on many of the points above.

Software and code

Policy information about availability of computer code

Data collection	JEOL ECS-400 (400 MHz) spectrometer, a Waters® Xevo® G2-XS Qtof high-resolution mass spectrometer coupled with an ACQUITY® UPLC® I-Class Bio System,<br>a Shimadzu Essentia Pre LC-16P (Essentia, Kyoto, Japan) equipped with a UV detector (SPD-16),<br>an Applied Biosystems 7900HT Fast Real-Time PCR System,<br>a PHS-3B digital pH meter
Data analysis	Clustal Omega and MEGA X were used for sequence alignment and making a phylogenetic tree.<br>Sigmaplot 12.5 was used for statistical analysis.<br>Delta™ and MestReNova 8.1.1(NMR), UNIFI and MassLynx MS Software (LC-HRMS).<br>ggplot2 and R package were used to deal with data visualization.

For manuscripts utilizing custom algorithms or software that are central to the research but not yet described in published literature, software must be made available to editors and reviewers. We strongly encourage code deposition in a community repository (e.g. GitHub). See the Nature Research guidelines for submitting code & software for further information.

Data

Policy information about availability of data

All manuscripts must include a data availability statement. This statement should provide the following information, where applicable:

- Accession codes, unique identifiers, or web links for publicly available datasets
- A list of figures that have associated raw data
- A description of any restrictions on data availability

Data supporting the findings of this study are available within the paper and its Supplementary Information files and are available from the corresponding author upon reasonable request. A reporting summary for this article is available as a Supplementary Information file. The source data underlying all figures are provided as a Source Data file. Genome sequence data of S. monomycini NRRL B-2409 that support the findings of this study have been deposited in the National Center for Biotechnology Information (NCBI) GenBank database with the BioProject accession code JAFIQY000000000 (https://www.ncbi.nlm.nih.gov/nuccore/JAFIQY000000000.1/).

Field-specific reporting

Please select the one below that is the best fit for your research. If you are not sure, read the appropriate sections before making your selection.

☒ Life sciences ☐ Behavioural & social sciences ☐ Ecological, evolutionary & environmental sciences

For a reference copy of the document with all sections, see nature.com/documents/nr-reporting-summary-flat.pdf

Life sciences study design

All studies must disclose on these points even when the disclosure is negative.

Sample size	Sample size was not predetermined by statistical method as this work did not involve animal models or human subjects. But, sample sizes with respect to number of readings taken or the number of independent experiments are indicated in the respective figure legends. For all assays sample size was determined in advance and was uniform.
Data exclusions	No data was excluded from the analysis.
Replication	Each experiment includes three independent replicates to conduct statistical analysis. All qPCR were repeated at least three times with different biological samples and led to similar results. All attempts at replication for all other assays were successful.
Randomization	Randomization is not relevant to our study ,as each of the engineered strains had a specific control or controls, to which it was directed prepared
Blinding	Blinding is not applicable in our study because there were no need to allocate samples into experimental groups. Experiment results are not subjective.

Reporting for specific materials, systems and methods

We require information from authors about some types of materials, experimental systems and methods used in many studies. Here, indicate whether each material, system or method listed is relevant to your study. If you are not sure if a list item applies to your research, read the appropriate section before selecting a response.

Materials & experimental systems

n/a	Involved in the study	
☒	☐	Antibodies
☒	☐	Eukaryotic cell lines
☒	☐	Palaeontology and archaeology
☒	☐	Animals and other organisms
☒	☐	Human research participants
☒	☐	Clinical data
☒	☐	Dual use research of concern

Methods

n/a	Involved in the study	
☒	☐	ChIP-seq
☒	☐	Flow cytometry
☒	☐	MRI-based neuroimaging
